# Meta-analysis of the accuracy for *RASSF1A* methylation in bronchial aspirates for the diagnosis of lung cancer

**DOI:** 10.1371/journal.pone.0299447

**Published:** 2024-07-25

**Authors:** Xu-ping Chen, Shi-xu He, Meng-you Chen, Fu-bin Chen, Peng Wu, Ping Shi, Shi-cai Zhao, Ling-yan Zhao, Xiao-min Xiong, Jia Zeng

**Affiliations:** 1 Department of Respiratory and Critical Care Medicine, Guangyuan Central Hospital, Guangyuan, Sichuan Province, China; 2 Intern, Qiqihar Medical University, Qiqihar, Heilongjiang Province, China; 3 Department of General Internal Medicine, Qianwei Xuefu Community Health Service Center, Leshan, SiChuan Province, China; Johns Hopkins University, UNITED STATES OF AMERICA

## Abstract

**Objective:**

To establish the diagnostic accuracy of *RASSF1A* (Ras association domain family 1 isoform) methylation using bronchial aspirates as an auxiliary method for diagnosing lung cancer through a systematic review and meta-analysis.

**Methods:**

Studies published prior to October 30, 2022, were retrieved from the Embase, PubMed, Web of Science, and Wan Fang databases using the keywords “lung cancer”, “*RASSF1A*”, “methylation”, and “bronchial aspirates”. A fixed or random effect model was used to calculate the combined sensitivity, specificity, positive likelihood ratios (LR), negative LR, diagnostic odds ratio (DOR), along with the respective 95% confidence intervals (CIs) and the area under the curve (AUC) with Q index. The threshold effect was defined by using the Spearman correlation coefficient, and the Deeks funnel plot was generated to evaluate publication bias.

**Results:**

Among the 12 trials that met the inclusion criteria, a total of 2388 participants were involved. The pooled results for the diagnosis of lung cancer were as follows, when compared to the pathological diagnosis: sensitivity of 0.47 (95% CI: 0.45–0.50), specificity of 0.96 (95% CI: 0.95–0.97), positive LR of 12.18 (95% CI: 8.96–16.55), negative LR of 0.56 (95% CI: 0.52–0.61), DOR of 24.05 (95% CI: 17.29–33.47), and AUC of 0.78 (Q index = 0.72), respectively. The sensitivity of the *RASSF1A* methylation assay was relatively low in a detailed subgroup analysis, fluctuating between 0.39 and 0.90, indicating a limitation in its diagnostic value for lung cancer. The *RASSF1A* methylation assay, on the other hand, demonstrated excellent specificity, suggesting a high exclusion value. Of note, the diagnostic sensitivity, specificity, DOR, and AUC for small cell lung cancer were 0.90 (0.84–0.94), 0.95 (0.94–0.97), 249.5 (103.94–598.8), and 0.98, respectively, showing that *RASSF1A* methylation was a promising biomarker for diagnosing small cell lung cancer with both high diagnostic and exclusion value. Furthermore, *RASSF1A* methylation using bronchial washings and bronchial aspirates showed a high AUC of 0.998 and 0.93, respectively, indicating excellent diagnostic performance.

**Conclusions:**

The methylation of *RASSF1A* in bronchial aspirates demonstrated a high level of diagnostic accuracy and has the potential to be a valuable supplementary diagnostic method, especially for identifying small cell lung cancer.

## Introduction

Lung cancer, which remains the leading cause of cancer-related deaths worldwide, is the most prevalent malignant tumor in humans [1]. Recent trends indicate that the incidence and mortality rates of lung carcinoma are increasing. In China, lung cancer ranked first in terms of incidence and mortality among malignant tumors with approximately 0.82 million new cases and about 0.72 million deaths in 2020 [2]. Lung cancer encompasses both non-small cell lung cancer (NSCLC) and small cell lung cancer (SCLC). The former, constituting about 85% of lung cancer cases, mainly comprises squamous cell carcinomas (SCC) and adenocarcinomas (AC) [3]. The high mortality rate among lung cancer patients persists chiefly due to over 75% of them being diagnosed in advanced stages, despite the progress made in new treatment options over time [4]. Genetic abnormalities have played a significant role in the development of lung cancer [5]. Hypermethylation of the promoter can also lead the loss of tumor-suppressor function, in addition to point mutations and deletions [6]. The most currently effective method for screening lung cancer is low-dose computed tomography (LDCT) [7]. However, when considering the risks of overdiagnosis, radiation exposure, and poor specificity that limit its diagnostic accuracy in LDCT screening, it becomes essential to develop specific and accurate screening tools in order to enhance the survival rates of lung cancer patients [8].

There is an increasing amount of evidence indicating that DNA methylation, a crucial molecular mechanism responsible for driving epigenetic alterations, might potentially be involved in the progression of lung cancer [9]. A rising number of methylation assays using either plasma or sputum have been proposed to diagnose lung cancer. Among these assays, the detection of *RASSF1A* hypermethylation is of particular interest [10]. As one of the three transcripts (transcripts A, B and C) of human *RAS* effector homologue (*RASSF1*) located in the 120-kb region of minimal homozygous deletion, transcript A (*RASSF1A*) which initiates at CpG island A is commonly expressed in all normal tissues [11]. *RASSF1A* (Ras association domain family 1 isoform) is related to the regulation of the cell cycle, migration, adhesion, and apoptosis [12]. Research has revealed that the loss of expression of *RASSF1A*, which may lead to tumorigenesis, is associated with hypermethylation of its CpG-island promoter sequence [13]. As one of the frequently reported genes in blood-based liquid biopsies, hypermethylation detection of *RASSF1A* has shown a sensitivity range of 22–66% and a specificity range of 57–100% in terms of detecting lung cancer [10]. In order to obtain superior specimens for histological or cytological examinations, fiberoptic bronchoscopy is currently considered a standard procedure for patients with suspected lung cancer. Due to its proximity to tumor cells, the bronchoalveolar lavage fluid (BALF) obtained through bronchoscopy is a highly valuable supplementary sample for the diagnosis of lung cancer [14]. Kim H et al. discovered that *RASSF1A* methylation assay on BALFs resulted in a diagnostic sensitivity of 37.6% and a specificity of 96.1% with a participation of 85 NSCLC patients, indicating that *RASSF1A* might be a potential biomarker for detection of NSCLC in bronchial lavages [15]. Liu JJ et al. however, found that the detection of hypermethylation in *RASSF1A* using BALFs from 60 patients with SCLC had a sensitivity of 86.7% and a specificity of 90%, which contradicted Kim’s findings [16]. It remains unknown whether the differences in results were caused by differences in lung cancer types or not.

To our knowledge, there has not been a comprehensive evaluation of the diagnostic accuracy of *RASSF1A* methylation on bronchial aspirates (BALFs and bronchial washings) for lung cancer diagnosis, despite the growing number of studies and meta-analyses that have examined the detection of *RASSF1A* hypermethylation using plasma or BALFs. Therefore, we conducted a systematic review and meta-analysis to assess the diagnostic accuracy of the *RASSF1A* promoter hypermethylation assay in diagnosing lung cancer using bronchial aspirates.

## Methods

### Search strategy

All studies published in the Embase, PubMed, Web of Science, and Wan Fang databases were independently searched by two researchers using the key terms “lung cancer”, “carcinoma of the lung”, “lung carcinoma”, “BALF”, “bronchoalveolar lavage fluid”, “bronchial washings (BWs)”, “bronchial aspirates”, “methylation”, and “*RASSF1A*”. The detailed search strategies were shown in S1 Table. All studies published until October 30, 2022, were taken into consideration. Two independent reviewers screened the literature, extracted the data, and analyzed the included studies for bias risk. When the opinions of the two researchers disagreed, a third researcher was consulted.

### Inclusion criteria and exclusion criteria

Prior to data extraction, the study’s inclusion and exclusion criteria were decided. The following inclusion criteria were applied: (i) the subjects were suspected or confirmed patients with lung cancer; (ii) the diagnosis of lung cancer was confirmed through histology or cytology; (iii) methylation assays for *RASSF1A* were conducted; (iv) BALF, bronchial washings, or bronchial aspirates were used as samples; (v) the data of the true positives (TP), false positives (FP), false negatives (FN), and true negatives (TN) was provided; and (vi) the full texts of all eligible studies were published either in the English or Chinese languages. The following criteria were included for exclusion: (i) duplicate publications or data; (ii) letters, abstracts, or conference abstracts; (iii) the genes involved were not *RASSF1A*; (iv) the methylation of *RASSF1A* was detected in plasma, sputum samples, and lung tissue; (v) incomplete data; and (vi) unrelated literature.

### Data extraction and methodological quality evaluation

The literature selection and data extraction were independently conducted by two researchers. Any inconsistencies were resolved through discussion. The extracted data included the following: first author; publication year; country; race; study design; assay methods; sample types; number of cases and controls; pathological types (S2 Table); tumor staging (S3 Table); primers and probes (S4 Table); test results involving TP, FP, FN, and TN; percentage of sensitivity and specificity; and any other relevant items for bias risk assessment. A two-person research team assessed each study’s risk of bias independently by using the Quality Assessment of Diagnostic Accuracy Studies 2 (QUADAS-2) tool [17]. A designation of “yes” (indicating low bias risk or good applicability), “no” (indicating high bias risk or poor applicability), or “unclear” (indicating a lack of relevant information or uncertainty of bias risk) was assigned to each item in order to assess the quality of each study.

### Statistical analysis

The software tools used for this meta-analysis were the Meta-Disc 1.4 (Clinical Biostatistics Unit, Madrid, Spain), Review Manager 5.4 (Cochrane Collaboration, Oxford, UK), and Stata 14.0 (Stata Corp LP). The sensitivity, specificity, positive likelihood ratio (LR), negative LR, diagnostic odds ratio (DOR) with 95% confidence intervals (CI), and the area under the curve (AUC) have been pooled. To determine whether to use a fixed or random effects model, one should consider the degree of heterogeneity. The heterogeneity was assessed using I^2^ statistics. The I^2^ was explicated as follows: 0–25%, indicating no heterogeneity; 26–50%, indicating low heterogeneity; 51–75%, indicating moderate heterogeneity; and >75%, indicating high heterogeneity. The threshold effect was defined by using the Spearman correlation coefficient, and a p value below 0.05 was deemed indicative of a threshold effect. Meta-regression and subgroup analyses were conducted utilizing seven independent variables, which include races (Asians and White), sample types (BALF, bronchial aspirates, and BWs), sample size (<100 and >100), assay methods (methylation-specific polymerase chain reaction [MSP], digital droplet MSP [ddMSP], and quantitative MSP [QMSP]), pathological types (AC, SCC, NSCLC, and SCLC), tumor staging (stage 0, I, II, III, and IV), and primers (A and B). Sensitivity analysis was utilized to evaluate the stability of pooled findings. A Deeks funnel plot was generated to assess publication bias and p<0.05 was deemed statistically significant.

## Results

### Publication searching and included studies

**Fig 1:**
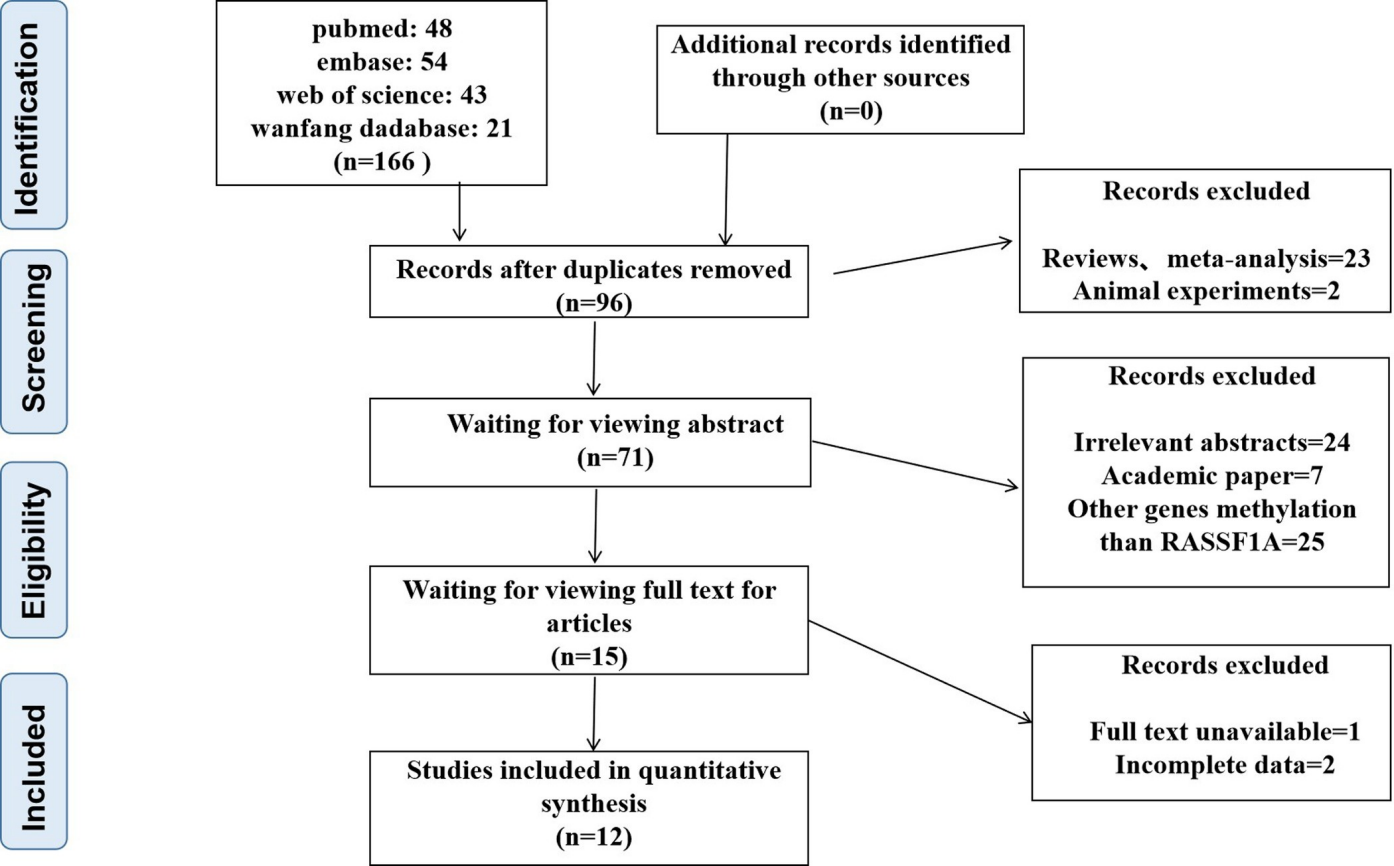
Flow chart depicting the process for selecting studies in this review.

In this meta-analysis, we included twelve studies with a total of 2388 subjects. 166 studies were selected from the search conducted on PubMed, Embase, Web of science, and Wanfang databases mentioned earlier. A total of 154 manuscripts were excluded for the following reasons: 25 were reviews, meta-analyses, or animal experiments; 56 were irrelevant abstracts, academic papers, or studies that focused on genes other than *RASSF1A*; and 3 with incomplete data or unavailable full texts. Finally, as shown in Fig 1, 12 eligible studies examining the methylation of the *RASSF1A* gene in bronchial aspirates from lung cancer patients were evaluated after removing duplicates and irrelevant records, based on the inclusion criteria.

### Characteristics and quality assessment of the included studies

In total, 1319 lung cancer patients and 1069 non-lung-cancer controls from 12 studies that investigated *RASSF1A* gene methylation were included in this meta-analysis [15,18–28]. There were 3 prospective studies and 9 retrospective studies. The pathological diagnosis was used as the reference standard in all 12 studies. Among the studies, 6 had sample types consisting of BALF, 3 were comprised of bronchial aspirates (BALF and BWs), and the remaining 3 were BWs alone. Out of all the studies that were included, 1 study utilized the ddMSP assay, 4 utilized the MSP assay, and 7 utilized the QMSP assay. Regarding the source of controls, 11 of the studies included controls from non-lung cancer patients, while 1 study used controls from non-cancerous tissue borders of lung cancer tissue. Table 1 presented the characteristics of the trials and participants that were included in the study (Table 1). Regarding primers, two papers utilized primer A, three papers utilized primer D, while the remaining papers utilized various primers (S4 Table).

**Table 1:** Summary of the diagnostic results of the included studies.

Study	Country	Race*	Case/control	Sample	Assay method	Study design	TP	FP	FN	TN	SEN (%)	SPE (%)
Ilse, P 2014 [18]	Germany	White	75/43	bronchial aspirates	QMSP	prospective	22	0	53	43	29.30	100.00
Grote HJ 2006 [25]	Germany	White	157/46	bronchial aspirates	QMSP	retrospective	78	0	79	46	49.70	100.00
Schmiemann V 2005 [24]	Germany	White	85/102	bronchial aspirates	QMSP	retrospective	35	0	50	102	41.20	100.00
Schramm M 2011 [22]	Germany	White	117/61	BWs	QMSP	prospective	58	1	59	60	49.60	98.30
van der Drift MA 2012 [21]	The Netherlands	White	129/28	BWs	QMSP	prospective	55	0	74	28	42.60	100.00
Roncarati R 2020 [26]	Italy	White	91/31	BWs	ddMSP	retrospective	42	0	49	31	46.20	100.00
Guo M 2004 [27]	Maryland	White	20/14	BALF	MSP	retrospective	6	0	14	14	30.00	100.00
Kim H 2004 [15]	Korea	Asians	85/127	BALF	MSP	retrospective	32	5	53	122	37.60	96.10
Ren M 2017 [23]	China	Asians	123/130	BALF	QMSP	retrospective	62	5	61	125	50.40	96.20
Chen RY 2019 [28]	China	Asians	131/145	BALF	MSP	retrospective	77	12	54	133	58.80	91.70
Zhang YM 2016 [19]	China	Asians	261/319	BALF	QMSP	retrospective	139	19	122	300	53.30	94.00
Yu ZT 2007 [20]	China	Asians	45/23	BALF	MSP	retrospective	19	0	26	23	42.20	100.00

**Abbreviations:** BALF: Bronchoalveolar lavage fluid; BWs: Bronchial washings; PCR: Polymerase chain reaction; MSP: Methylation-specific PCR; QMSP: Quantitative methylation-specific PCR; ddMSP: Droplet digital MSP; SEN: Sensitivity; SPE: Specificity. *The race was deduced by the country of study which might not reflect the truth or the race of participants.

**Fig 2:**
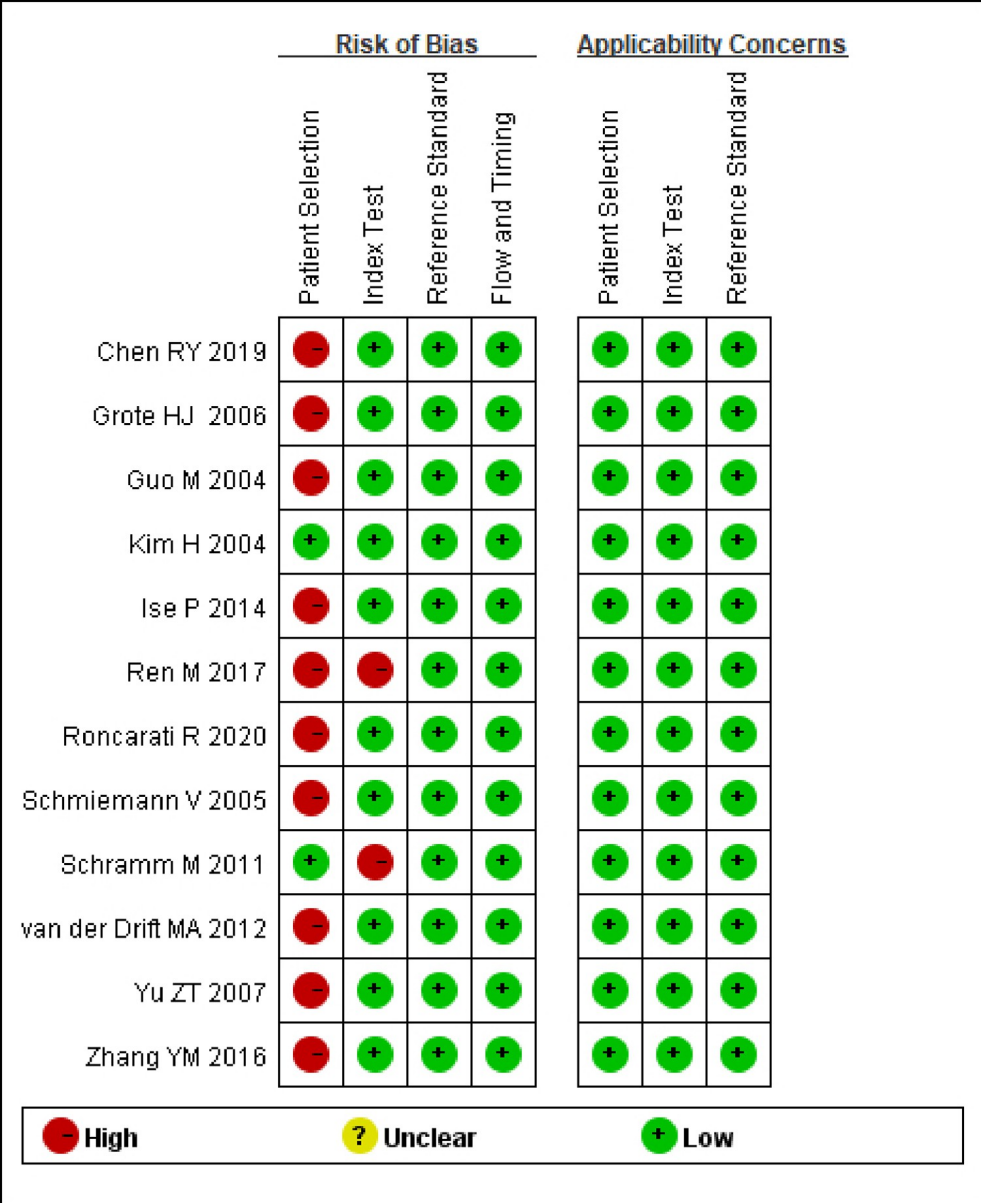
Bias risks and applicability concerns.

According to Fig 2, the 12 included studies were evaluated for their quality and applicability. In terms of patient selection, 2 studies had a low risk of bias, while the remaining 10 were considered “high” due to unclear recruitment procedures and the failure to avoid a case-control study design. Regarding index testing, 2 studies were classified as having a “high risk of bias” due to performing the trials with prior knowledge of the gold standard test results, while the remaining ones were labeled as “low risk”. When considering the involvement of patients in the analysis of applicability and reference standards, all biases and concerns related to their applicability were rated as “low”.

### Meta-analysis results of the *RASSF1A* methylation

**Fig 3:**
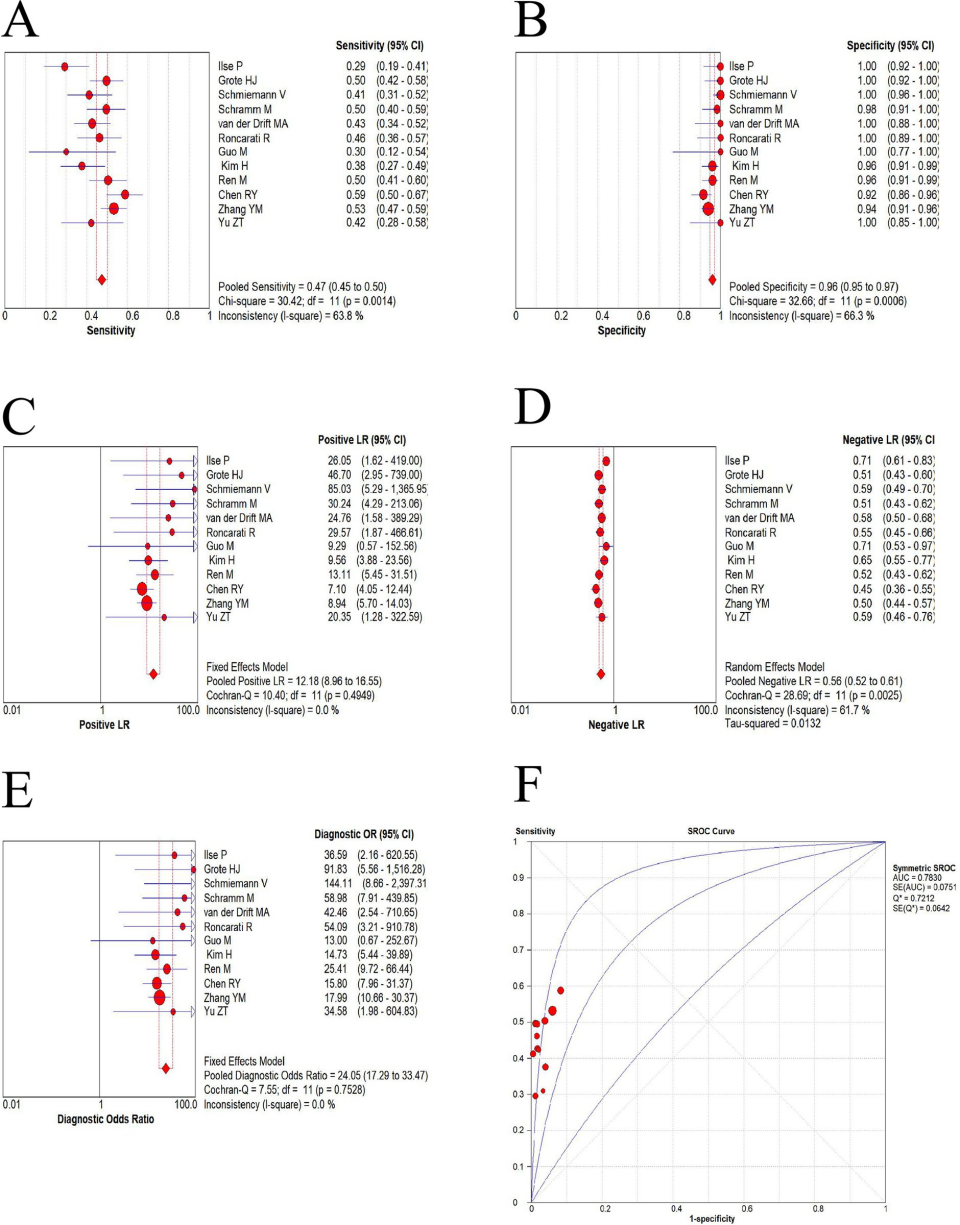
The pooled sensitivity, specificity, positive LR, negative LR, DOR, and AUC of *RASSF1A* methylation assays using bronchial aspirates for diagnosing lung cancer. The diamond represents the combined values, while the lines represent the corresponding 95% confidence intervals (CI).

The aim of this study was to assess the diagnostic potential of *RASSF1A* methylation status using bronchial aspirates to assist in the diagnosis of lung cancer. When using *RASSF1A* methylation assays of bronchial aspirates from lung cancer as a comparison to non-lung cancer controls or non-cancerous borders of lung cancer tissues, the results showed a combined sensitivity of 0.47 (95% CI: 0.45–0.50), specificity of 0.96 (95% CI: 0.95–0.97), positive LR of 12.18 (95% CI: 8.96–16.55), negative LR of 0.56 (95% CI: 0.52–0.61), DOR of 24.05 (95% CI: 17.29–33.47), and AUC of 0.78 (Q index = 0.72), respectively. The combined positive LR and DOR were calculated using a fixed-effects model, while the pooled sensitivity, specificity, negative LR, and the AUC were computed using a random-effects model. The SROC (summary receiver operating characteristic) curve and forest plots for sensitivity, specificity, positive LR, negative LR, and DOR of *RASSF1A* methylation assays in lung cancer diagnosis from the included studies were shown in Fig 3. It is worth noting that there was a significant heterogeneity (I^2^ > 50%) in the combined sensitivity, specificity, and negative LR across the studies. To determine the cause, this heterogeneity was further investigated using meta-regression and subgroup analysis.

### Meta-regression and subgroup analyses of the sensitivity and specificity for the *RASSF1A* methylation in lung cancer

A meta-regression analysis revealed consistent values in sensitivity across subgroups that were based on different races, sample types, sample size, assay methods, tumor staging, and primers (p>0.05). However, the sensitivity for the SCLC subgroup (0.90 [0.84–0.94]) was significantly higher than that for the NSCLC subgroup (0.41 [0.37–0.45]) (p<0.001). Additionally, the higher the grade of the tumor stage, the higher the pooled sensitivity, except for stage IV. Furthermore, there were variations in specificity values among subgroups categorized by races, sample types, sample size, and primers (p<0.05); however, these values remained consistent across subgroups categorized by assay methods, pathological types, and tumor staging (Table 2).

**Table 2:** The sensitivity and specificity subgroup analyses of *RASSF1A*: Different races, sample types, sample size, assay methods, pathological types, tumor staging, and primers.

Parameter (no.of studies	AUC	Sensitivity (95%CI)	p		Specificity (95%CI)	p	
**Race**							
Asians(5)	0.85	0.51(0.47–0.55)	>0.05		0.95(0.93–0.96)	<0.01	
White(7)	0.75	0.44(0.40–0.48)			0.997(0.98–1.00)		
**Sample type**							
BALF(6)	0.86	0.50(0.47–0.54)	>0.05		0.95(0.93–0.96)	<0.05	
BWs(3)	0.998	0.46(0.41–0.52)			0.99(0.95–1.00)		
Bronchial aspirates(3)	0.93	0.43(0.37–0.48)			1.00(0.98–1.00)		
**sample size**							
>100(10)	0.78	0.48(0.45–0.51)	>0.05		0.96(0.95–0.97)	<0.01	
<100(2)	0.50	0.39(0.27–0.51)			1.00(0.91–1.00)		
**Assay method**							
MSP(4)	0.87	0.48(0.42–0.54)	>0.05		0.95(0.91–0.97)	>0.05	
QMSP(7)	0.71	0.47(0.44–0.51)			0.97(0.95–0.98)		
ddMSP(1)	NA	0.46(0.36–0.57)			1.00(0.89–1.00)		
**Pathological types**							
AC(6)	0.64	0.42(0.37–0.47)	>0.05		0.95(0.94–0.97)	>0.05	
SCC(6)	0.67	0.39(0.33–0.46)			0.95(0.94–0.97)		
NSCLC(6)	0.61	0.41(0.37–0.45)	<0.001		0.95(0.94–0.97)	>0.05	
SCLC(6)	0.98	0.90(0.84–0.94)			0.95(0.94–0.97)		
**Tumor staging**							
Stage 0(2)	0.5	0.44(0.14–0.79)		>0.05	0.94(0.90–0.96)		>0.05
Stage I(3)	0.5	0.51(0.38–0.63)	>0.05		0.94(0.90–0.96)	>0.05	
Stage II(3)	0.5	0.51(0.36–0.66)			0.94(0.90–0.96)		
Stage III(3)	0.77	0.62(0.49–0.74)	>0.05		0.94(0.91–0.97)	>0.05	
Stage IV(3)	0.87	0.59(0.46–0.71)			0.94(0.91–0.97)		
**Primers**							
Primer A(2)		0.47(0.40–0.53)	>0.05		1.00(0.98–1.00)	<0.05	
Primer D(3)		0.54(0.50–0.58)			0.94(0.92–0.96)		

**Abbreviations:** Adenocarcinoma, AC; squamous carcinoma, SCC; Non-small cell lung cancer, NSCLC; Small cell lung cancer, SCLC.

Taken together, our data showed slight fluctuations in the sensitivity and specificity across subgroups. In all subgroups of *RASSF1A* methylation, the sensitivity has commonly been low, except for the SCLC subgroup, while specificity was generally high, particularly in the subgroups of white individuals, bronchial aspirates, sample size of less than 100, QMSP, and primer A (Table 2).

### Diagnostic accuracy analysis

The SROC curve for *RASSF1A* promoter methylation assays in the diagnosis of lung cancer and the AUC values for each subgroup were presented in Fig 3 and Table 2, respectively. According to the results of the SROC curve, the AUC for *RASSF1A* methylation was 0.78, with a Q index of 0.72. The subgroup analysis revealed that the AUC values for the subgroups of BWs, bronchial aspirates, and SCLC were 0.998, 0.93 and 0.98, respectively. This confirmed that the detection of *RASSF1A* methylation holds a significant degree of accuracy in diagnosing lung cancer, particularly when utilizing BWs or bronchial aspirates, and in cases of SCLC.

### Threshold analysis

For *RASSF1A*, the Spearman correlation coefficient was 0.406, with a p-value of 0.191 (p>0.05). No threshold effect was observed.

### Sensitivity analyses

**Fig 4:**
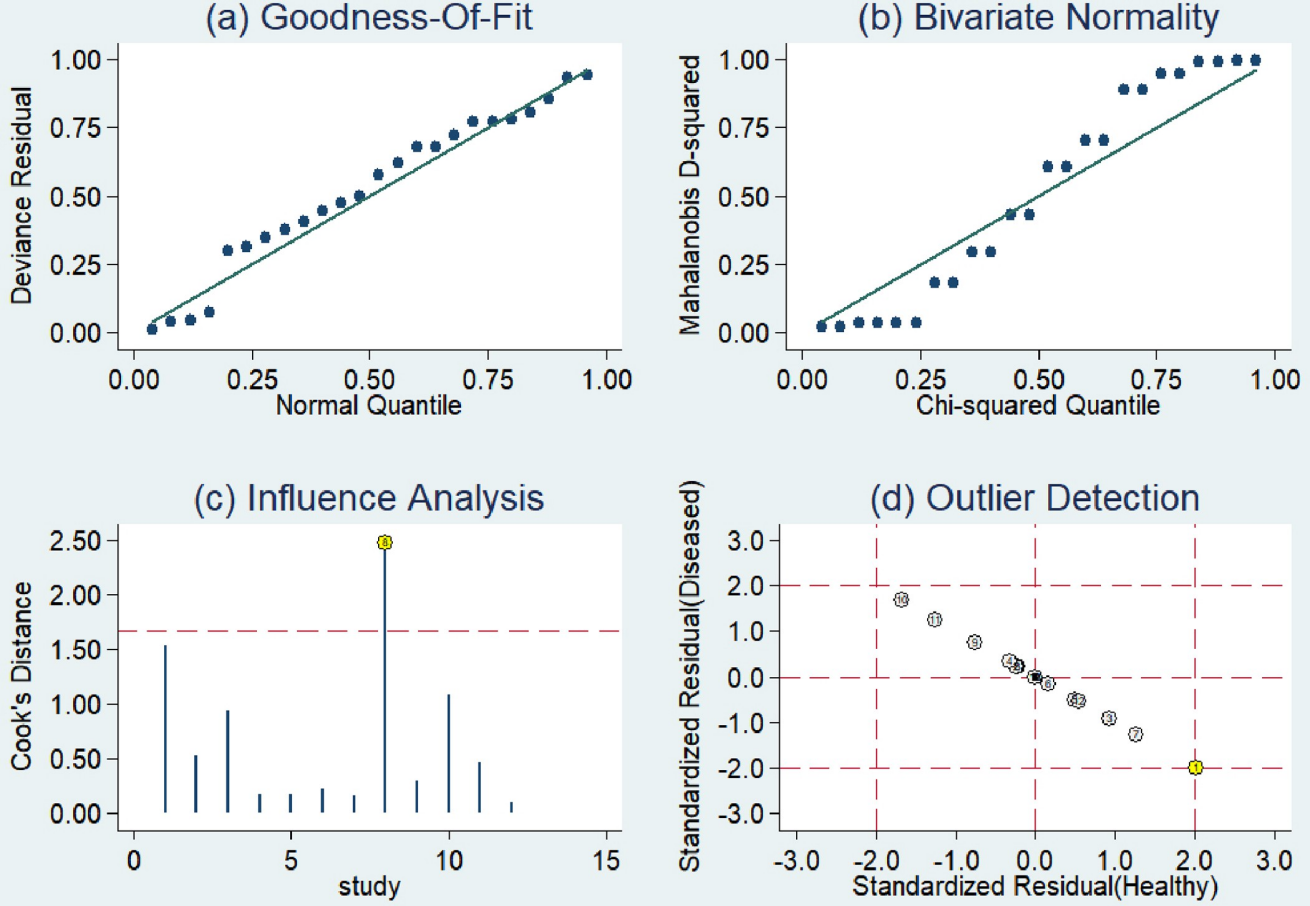
The sensitivity analyses for *RASSF1A* methylation.

It was evident from Fig 4 that there was 1 original study (Kim H, 2004) [15] with a strong influence on sensitivity, while the other original studies did not impact the calculation results. In general, the findings of this study displayed a relatively consistent pattern.

### Publication bias

**Fig 5:**
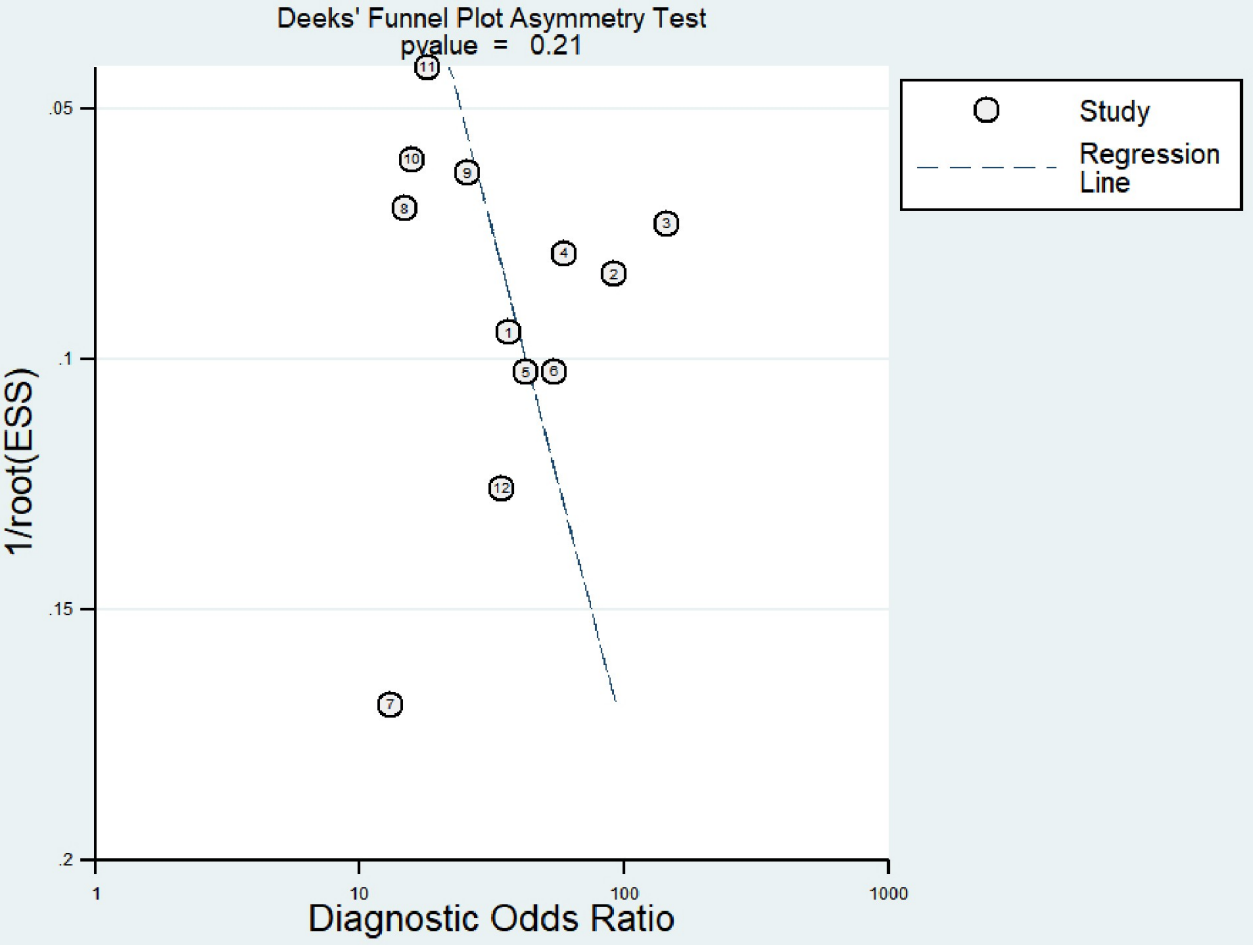
The Deeks funnel plot for *RASSF1A* methylation.

According to the Deeks funnel plot, *RASSF1A* did not show any publication bias, as indicated by a p-value of 0.21 (p>0.05) (Fig 5).

## Discussion

Aberrant *RASSF1A* promoter methylation assays using serum or BALFs has been reported to be a promising detection as a diagnostic adjunct for clinically suspected lung carcinoma and its surveillance [29]. However, the hypermethylation status of *RASSF1A* on bronchial aspirates has not yet been examined in cases of lung cancer. In order to evaluate whether the utilization of *RASSF1A* hypermethylation assay using bronchial aspirates could be a viable complementary method for screening lung cancer or not, a systematic analysis of research publications was conducted.

Research findings on the utilization of bronchial aspirates to diagnose lung cancer through *RASSF1A* methylation have yielded inconsistent results. This meta-analysis revealed that the diagnostic sensitivity, specificity, DOR, and AUC were 0.47 (0.45–0.50), 0.96 (0.95–0.97), 24.05 (17.29–33.47), and 0.78, respectively. It was noteworthy that the pooled results showed remarkably high specificity, indicating an especially high exclusion value for lung cancer diagnosis. However, the sensitivity was relatively low, which could restrict its use as a lung cancer screening tool in clinical settings. In any case, one must exercise caution when interpreting the data, particularly in the context of positive cases involving *RASSF1A* methylation.

There was significant heterogeneity in the sensitivity and specificity of the studies. Of note, heterogeneity can affect the accuracy of the meta-analysis results, thereby impacting their validity. Heterogeneity in diagnostic tests meta can be attributed to threshold effects as well as non-threshold effects. Due to the use of different diagnostic cut-off values in the included studies, threshold effects may occur, while factors such as gender and age could lead to non-threshold effects. The spearman correlation coefficient was applied to examine whether there was a threshold effect in this meta-analysis. The results demonstrated that no threshold effect, stemming from variations in diagnostic cut-offs among studies, was observed. Meta-regression and subgroup analyses were further performed to explore sources of heterogeneity. The results showed that the sensitivity of *RASSF1A* methylation detection remained inconsistent across subgroups of pathological types, and the specificity varied across subgroups based on races, sample types, and sample size. Generally, the sensitivity was low, fluctuating between 0.38 and 0.62, indicating that its diagnostic value for lung cancer was limited. However, the diagnostic sensitivity, specificity, DOR, and AUC for SCLC were 0.90 (0.84–0.94), 0.95 (0.94–0.97), 249.5 (103.94–598.8), and 0.98, respectively, showing that *RASSF1A* methylation may be a promising biomarker for SCLC diagnosis (sensitivity = 0.90>0.5, specificity = 0.95>0.90, DOR = 249.5, and AUC = 0.98>0.90) with both extremely high diagnostic and exclusion diagnostic value. In comparations between SCLC and NSCLC, we discovered a correlation between *RASSF1A* methylation and these two histotypes, indicating the methylation status of the *RASSF1A* gene was associated with lung cancer histology. The data indicated that *RASSF1A* methylation has the potential to be useful in differentiating between SCLC and NSCLC, as SCLC exhibited a considerably higher level of methylation compared to NSCLC (p<0.001). Furthermore, the sensitivity increased as the grade of the tumor stage increased, except for stage IV, although this difference was not statistically significant. Research has revealed that SCLC or advanced lung cancer are more likely to exhibit higher levels of aggressiveness, faster growth, increased development of neoplastic necrosis, and more pronounced symptoms as the tumor stage increases. This, in turn, facilitates the easier detection of methylation due to the heightened release of DNA [30,31]. Meanwhile, Dammann, R et al. discovered that *RASSF1A* was not expressed in any of the SCLC cell lines, as well as several other cancer cell lines that were examined [13].

The *RASSF1A* methylation assay, however, demonstrated excellent specificity, ranging from 0.92 to 1.00, indicating a high exclusion value. Subgroups of bronchial aspirates, white individuals, sample size of less than 100, and primer A exhibited higher specificity compared to their counterparts (p<0.05). It was speculated whether bronchial aspirates contain higher concentrations of tumor cells than BALF, resulting in higher specificity or not [32]. Furthermore, QMSP, which was more specific than MSP in detecting *RASSF1A* methylation in subgroup analysis, was predominantly utilized for detecting *RASSF1A* hypermethylation in studies involving white individuals and primer A. The quantitative methylation-specific real-time PCR (QMSP), also referred to as methylight, is a highly specific method that offers the advantage of reducing the potential for non-specific amplification. This is achieved by incorporating a TaqMan probe alongside conventional methylation-specific primers [33]. The reasons why the subgroup with a sample size of less than 100 was more specific than that of more than 100 cannot be easily explained by factors such as races, detection methods, pathological classifications, specimen types, or tumor staging. However, this work suggests that it may be a factor, and some studies included a small sample size, thus requiring careful interpretation of the results.

Overall, the results of this study remained relatively stable from the sensitivity analysis. However, one of the primary objectives of the meta-analysis was to investigate the factors contributing to the heterogeneity. Our analysis has revealed significant heterogeneity, and the spearman approach has demonstrated that this heterogeneity cannot be explained by threshold effects. According to a detailed subgroup analysis, it remains to be elucidated through further investigations with well-designed studies and long-term follow-up on patients whether the heterogeneity is primarily due to race, sample type, sample size, pathological types of lung cancer, and primers or not.

In terms of diagnostic yield, hypermethylation assays offer advantages over conventional assay methods, such as histology or cytology, particularly when considering the presence of false-negative biopsies in the diagnosis of lung cancer. According to Schmiemann V, et al, *RASSF1A* hypermethylation assays performed with QMSP on bronchial aspirates were correctly confirmed in 88% and 25% of cases with highly doubtful or suspicious cytology, respectively, and this technique proved to be a rather helpful diagnostic tool in cases of peripheral lung cancers where simultaneous cytology and histology tests yielded negative results [24]. Nevertheless, the detection of *RASSF1A* methylation relies on the accessibility of sophisticated equipment and incurs significant expenses; moreover, bronchial aspirates must be obtained through invasive procedures, posing challenges for the elderly and individuals with comorbidities. Therefore, the clinical application of this assay is limited. It is worth noting that not all of the literature included provided information on the primers used and the precise CpGs they cover. That information would be very helpful for reproducing and validating the data. Consequently, in this analysis, we are not comparing the specific region of the *RASSF1A* promoter analyzed by each paper. We are pointing out what was described and are aware that this might be a limitation, due to the fact that the different tools might be analyzing different regions of the *RASSF1A* gene. Therefore, we are unable to determine whether different locations of CpGs lead to varying sensitivities. This meta-analysis still has the following limitations: (i) Due to limitations in study design, not all of the included studies possessed high methodological quality; (ii) There was still significant heterogeneity with an unknown source; (iii) some subgroups, such as those with a sample size of less than 100 and utilizing the ddMSP assay method, were included in only a very limited number of studies; (iv) The majority of the studies included were retrospective studies conducted at a single institution; (v) None of the literature included provided information on the ethnicity of the patients and the race information in Table 1 came from the country where the article was performed. Therefore, subgroup analysis that includes race could potentially be biased to some extent; (vi) Data regarding age, gender, smoking status, tumor location, and percentage of tumor cells within the identified tissue were unavailable; and (vii) No unpublished literature were included in this study. The analysis may have been somewhat biased due to these.

## Conclusions

In conclusion, this meta-analysis found that *RASSF1A* methylation testing using bronchial aspirates provides a strong diagnostic ability, particularly in excluding lung cancer, and has the potential to be used as a complementary diagnostic tool. It is noteworthy that the *RASSF1A* methylation assay on bronchial aspirates demonstrated high sensitivity and specificity in identifying SCLC, suggesting its exceptional diagnostic and exclusionary utility. However, the current overall accuracy of the test is insufficient to be utilized for diagnosing lung cancer in clinical settings. Therefore, well-designed multicenter and prospective large-scale studies are necessary to confirm the diagnostic utility of the *RASSF1A* promoter hypermethylation assay using bronchial aspirates for detecting lung cancer.

## Supporting information

S1 Checklist PRISMA 2009 checklist. Checklist showing what page are each characteristic analyzed.
(DOCX)

S1 Table Search strategies for this meta-analysis. (XLSX)

S2 Table Summary of the diagnostic results for different lung cancer types of the included studies. (XLSX)

S3 Table Summary of the diagnostic results for different tumor stages of the included studies. (XLSX)

S4 Table Summary of the primers of the included studies. (XLSX)

S1 File Four pdfs for Chinese references including Liu JJ 2021 [16], Chen RY 2019 [28], Yu ZT 2007 [20], and Zhang YM 2016 [19]. (ZIP)
