# Supplementary material for: Meta-analysis of the accuracy for RASSF1A methylation in bronchial aspirates for the diagnosis of lung cancer
Source: PLoS One. 2024 Jul 25;19(7):e0299447. doi: 10.1371/journal.pone.0299447 (PMC11271935; doi:10.1371/journal.pone.0299447)
Supplement: S1 Checklist — Checklist showing what page are each characteristic analyzed. (DOCX) [file pone.0299447.s001.docx]

| **Section and Topic** | **Item #** | **Checklist item** | **Location where item is reported** |
| --- | --- | --- | --- |
| **TITLE** | | |  |
| Title | 1 | The report is identified as a meta-analysis. | 1 |
| **ABSTRACT** | | |  |
| Abstract | 2 | The structured abstract includes objective, methods, results and conclusion. | 1-2 |
| **INTRODUCTION** | | |  |
| Rationale | 3 | Described in the introduction. | 2-4 |
| Objectives | 4 | Stated in the introduction. | 2-4 |
| **METHODS ,** | | |  |
| Eligibility criteria | 5 | Specified in the methods. | 5 |
| Information sources | 6 | Specified in the methods. | 4 |
| Search strategy | 7 | Present in supporting Information (file of search strategies). | File of search strategies |
| Selection process | 8 | Specified in the methods. | 5 |
| Data collection process | 9 | Specified in the methods. | 5 |
| Data items | 10a | Described in the methods. | 5-6 |
|  | 10b | Described in the methods. | 5-6 |
| Study risk of bias assessment | 11 | Specified in the methods. | 5-6 |
| Effect measures | 12 | Specified in the methods. | 6 |
| Synthesis methods | 13a | Described in the Statistical analysis of methods. | 6 |
|  | 13b | Described in the Statistical analysis of methods. | 6 |
|  | 13c | Described in the Statistical analysis of methods. | 6 |
|  | 13d | Described in the Statistical analysis of methods. | 6 |
|  | 13e | Described in the Statistical analysis of methods. | 6 |
|  | 13f | Described in the methods. | 6 |
| Reporting bias assessment | 14 | Specified in the methods. | 5 |
| Certainty assessment | 15 | Described in the methods. | 5 |
| **RESULTS** | | |  |
| Study selection | 16a | Described in the results. | 7 |
|  | 16b | Described in the results. | 7 |
| Study characteristics | 17 | Described in the results. | 7 |
| Risk of bias in studies | 18 | Presented in the results. | 7 |
| Results of individual studies | 19 | Presented in the text description section of results and table 1. | 7 |
| Results of syntheses | 20a | Summarised in the results. | 7-8 |
|  | 20b | Presented in the results. | 8 |
|  | 20c | Meta-regression and analysis of subgroups were done which were presented in the results. | 8-10 |
|  | 20d | Presented in the Sensitivity analyses part of the results. | 10 |
| Reporting biases | 21 | Presented in the publication bias of the results. | 10 |
| Certainty of evidence | 22 | Presented in the results. | 8-9 |
| **DISCUSSION** | | |  |
| Discussion | 23a | Provided in the discussion part. | 10-13 |
|  | 23b | Discussed in the discussion. | 13-14 |
|  | 23c | Discussed in the discussion. | 13-14 |
|  | 23d | Discussed in the discussion. | 14 |
| **OTHER INFORMATION** | | |  |
| Registration and protocol | 24a | The protocol is described in the Methods. Registration does not apply. |  |
|  | 24b | The protocol is described in the Methods. Registration does not apply. |  |
|  | 24c | The protocol is described in the Methods. Registration does not apply. |  |
| Support | 25 | Described in the part of acknowledgements. | 15 |
| Competing interests | 26 | Declared in the part of conflicts of interest. | 15 |
| Availability of data, code and other materials | 27 | Reported in the table 1. | Table 1 |

*From:*  Page MJ, McKenzie JE, Bossuyt PM, Boutron I, Hoffmann TC, Mulrow CD, et al. The PRISMA 2020 statement: an updated guideline for reporting systematic reviews. BMJ 2021;372:n71. doi: 10.1136/bmj.n71

For more information, visit: <http://www.prisma-statement.org/>
