# Supplementary material for: Meta-analysis of the accuracy for RASSF1A methylation in bronchial aspirates for the diagnosis of lung cancer
Source: PLoS One. 2024 Jul 25;19(7):e0299447. doi: 10.1371/journal.pone.0299447 (PMC11271935; doi:10.1371/journal.pone.0299447)
Supplement: S1 File — (ZIP) [file pone.0299447.s006.zip › S1 File/Chen RY 2019.pdf]

doi:10.13705/j.issn.1671-6825.2019.03.005

支气管肺泡灌洗液中 SHOX2 和 RASSF1A 基因甲基化检测对肺癌的诊断价值

陈瑞英[1)],刘　雅[2)],孙　婷[1)],刘峰辉[1)],马小花[1)]

1)郑州大学第一附属医院呼吸及睡眠科 郑州 450052　2)郑州大学第五附属医院呼吸科 郑州 450052

关键词　肺癌;DNA 甲基化;SHOX2; RASSF1A

中图分类号　R734.1

摘要　**目的**:对疑诊肺癌患者支气管肺泡灌洗液(BALF)中 SHOX2、RASSF1A 基因甲基化分析,评估两指标联合检测区分肺癌与肺良性疾病的诊断效能及联合细胞学使用对肺癌的诊断价值。**方法**:收集 276 例肺部疾病患者(肺癌组 131 例,肺部良性疾病组 145 例)的 BALF 样本,应用 PCR 法检测 BALF 中 SHOX2 和 RASSF1A 基因甲基化,并将检测结果和 Sanger 测序法结果比较。**结果**:PCR 和 Sanger 测序法一致性分析显示两种方法在检测 SHOX2 和 RASSF1A 基因甲基化具有良好的一致性(*Kappa* = 0.978, 95% *CI* = 0.954 ~ 1.000)。SHOX2 和 RASSF1A 基因甲基化联合检测在肺癌组的诊断灵敏度为 0.840,高于良性疾病组(0.193)($P < 0.001$);肺癌组 BALF 中 SHOX2、RASSF1A 基因甲基化联合检测阳性率在小细胞肺癌和肺鳞癌中分别是 100% 和 90.9%,高于肺腺癌(66.0%);SHOX2 和 RASSF1A 基因甲基化联合检测阳性率在晚期肺癌高达 89.3%,明显高于早期肺癌阳性检出率。ROC 曲线分析显示当 SHOX2、RASSF1A 基因甲基化、细胞学三指标联合使用时进一步将诊断灵敏度提高至0.931。**结论**:对 BALF 中 SHOX2、RASSF1A 基因甲基化 PCR 检测在肺癌诊断中有良好的灵敏度;SHOX2、RASSF1A 甲基化 PCR 检测与细胞学联合使用可显著提高肺癌诊断灵敏度,是肺癌病理学诊断的有效补充工具。

Value of DNA methylation analysis of SHOX2 and RASSF1A in bronchoalveolar lavage fluid in diagnosis of lung cancer

CHEN Ruiying[1)], LIU Ya[2)], SUN Ting[1)], LIU Fenghui[1)], MA Xiaohua[1)]

1) Department of Respiration and Sleep Medicine, the First Affiliated Hospital, Zhengzhou University, Zhengzhou 450052　2) Department of Respiration, the Fifth Affiliated Hospital, Zhengzhou University, Zhengzhou 450052

Keywords　lung cancer; DNA methylation; SHOX2; RASSF1A

Abstract　Aim: To analyze the methylation of SHOX2 and RASSF1A genes in bronchoalveolar lavage fluid(BALF) of patients with suspected lung cancer, and to evaluate the diagnostic efficacy of combination of two indicators to distinguish

【基金项目】河南省科技攻关项目(172102310087)

【作者简介】陈瑞英,女,1971 年 5 月生,硕士,副教授,副主任医师,研究方向:肺癌发病机制,E-mail:ring0008@163.com

hypertrophy induced by tumor necrosis factor-α[J]. Am J Transl Res,2017,9(2):343

[13] HU Hai, TIAN MX, DING C, et al. The C/EBP homologous protein (CHOP) transcription factor functions in endoplasmic reticulum stress-induced apoptosis and microbial infection[J]. Frontiers Immunol,2019,9:3083

[14] HETZ C, RUSSELAKIS-CARNEIRO M, MAUNDRELL K, et al. Caspase-12 and endoplasmic reticulum stress mediate neurotoxicity of pathological prion protein[J]. EMBO J, 2003,22(20):5435

[15] CHEN Y, TANG Y, XIANG Y, et al. Shengmai injection improved doxorubicin-induced cardiomyopathy by alleviating myocardial endoplasmic reticulum stress and Caspase-12 dependent apoptosis[J]. Biomed Res Int, 2015. doi: 10.1155/2015/952671

(2018-07-03 收稿　责任编辑赵秋民)

万方数据

lung cancer from benign lung disease and the diagnostic value of combined cytology for lung cancer. **Methods**: The BALF samples of 276 patients with lung diseases(131 patients with lung cancer, 145 patients with benign lung lesion) were collected and the methylation of SHOX2 and RASSF1A genes in BALF were detected by PCR. The results were compared with the results of Sanger sequencing. **Results**: Consistency analysis of PCR and Sanger sequencing showed that the two methods were in good agreement for detecting SHOX2 and RASSF1A gene methylation($Kappa = 0.978$, 95% $CI = 0.954 - 1.000$). The combined sensitivity of SHOX2 and RASSF1A gene methylation in the lung cancer group was 0.840, which was significantly higher than that in the control group(0.193) ($P < 0.001$). The positive rate of combined detection of SHOX2 and RASSF1A gena methylation in BALF was 100% and 90.9% in small cell lung cancer and squamous cell lung cancer respectively, higher than that(66.0%) in lung adenocarcinoma; The positive rate of combined detection of SHOX2 and RASSF1A gena methylation was 89.3% in advanced lung cancer, higher than early lung cancer. ROC curve analysis showed that when the SHOX2, RASSF1A gene methylation and cytology were used in combination, the diagnostic sensitivity was further increased to 0.931. **Conclusion**: The methylation analysis of SHOX2 and RASSF1A genes in BALF by PCR has good sensitivity and specificity in the diagnosis of lung cancer; SHOX2, RASSF1A methylation analysis by PCR combined with cytology can significantly improve the sensitivity of lung cancer diagnosis, and is an effective supplement for pathological diagnosis of lung cancer.

提高肺癌诊断率并及早治疗是降低肺癌高死亡率的关键[1-2]。在美国,低剂量计算机断层扫描(low-dose computed tomography, LDCT)筛查已被证明可降低高危肺癌患者的死亡率,但LDCT筛查存在假阳性及过度诊断的问题[3]。对疑似肺癌患者,通过纤维支气管镜获取活组织检查是确诊肺癌常用的侵袭性方法,但其诊断阳性率受病灶部位、大小、肺部合并症等因素影响,大约50%患者首次支气管镜检呈阴性结果[4]。支气管肺泡灌洗液(bronchoalveolar lavage fluid,BALF)细胞学检查一定程度上不受病灶位置限制,但受人为因素影响大,灵敏度低(43%~48%)。因此,需要探索一种高度敏感、特异的BALF分析方法作为病理学检查的补充,以提高肺癌的诊断率。

DNA甲基化是表观遗传学研究的重要方面,其与恶性肿瘤的发生、发展密切相关,参与了细胞癌变过程中的早期事件[5]。随着以甲基化特异性PCR(methylation-specific PCR, MS-PCR)为代表的检测技术的发展,人们发现多种基因的甲基化状态与肺癌关系密切[6-8]。矮小同源盒基因2(short stature homobox 2,SHOX2)是目前肺癌诊断中研究较多的甲基化指标,无论单独还是与组织/细胞学检查联合应用,可以有效提高肺癌检出率[9-11];研究[10,12]还显示,不同肺癌病理类型中存在组织特异性甲基化水平改变。Ras相关区域家族蛋白1A(ras-association domain family 1A, RASSF1A)被认为是新型抑癌基因,与多种恶性肿瘤的发生密切相关。研究[13-14]发现,90%以上的小细胞肺癌及50%~80%的非小细胞肺癌RASSF1A呈杂合性或纯合性缺失,RASSF1A在肺癌中存在着较高的表达。本研究测定了可疑肺癌患者BALF中SHOX2和RASSF1A基因甲基化状态,评估其在肺癌诊断中的应用价值。

万方数据

1 对象与方法

1.1 研究对象 研究对象均来自于郑州大学第一附属医院,研究方案经该院伦理委员会同意并与患者签署知情同意书。收集2015年1月至2017年6月在该院就诊、经胸部影像学检查发现肺部高密度阴影病灶或有其他肺部症状(咳嗽、咯血、胸闷、胸腔积液等)的疑似肺癌患者276例,进一步经组织病理学和(或)BALF细胞学确诊,原发性肺癌患者131例(病理类型:肺腺癌47例,鳞癌44例,小细胞肺癌31例,大细胞肺癌5例,其他类型4例;分期:Stage 0期5例,Ⅰ期18例,Ⅱ期32例,Ⅲ期39例,Ⅳ期28例,分期未明9例),肺部良性疾病145例。肺癌组年龄(59.15±13.25)岁,肺部良性疾病组年龄(60.56±13.45)岁,两组年龄差异无统计学意义($t = 0.878$, $P = 0.381$)。肺癌组男性患者91例,女性40例;肺部良性疾病组男性患者115例,女性30例,性别构成差异无统计学意义($\chi^2 = 1.041$, $P = 0.308$)。

1.2 BALF留取与病理分析 276例患者均接受纤维支气管镜检查,操作符合2013年英国胸科协会成人纤支镜检操作规范[15]。在病变肺段注入37 ℃灭菌生理盐水50 mL,立即用负压吸引回收灌洗液,共灌洗2次,记录回收的BALF总量(每位患者回收量不低于40 mL);然后进行支气管活检,采集组织学标本。

标本处理:取20 mL BALF,2 000 r/min离心10 min,去上清,用20~25 mL蒸馏水重悬沉淀,再次以

2 000 r/min 离心 10 min,去上清,取沉淀制作乙醇固定玻片并行 Papanicolaou 染色,进行细胞学检查。固定支气管活检或手术标本,并用苏木精-曙红染色,进行组织病理学检查。由该院资深病理学家阅片并出具诊断报告。

1.3　BALF 中 SHOX2 和 RASSF1A 基因甲基化检测

1.3.1　DNA 提取和处理　取 10 mL BALF,以 10 000 r/min 离心 5 min,取沉淀用于 DNA 提取。使用标准试剂盒(北京天根生化科技有限公司)提取基因组 DNA,提取后的 DNA 直接进行亚硫酸盐修饰,具体步骤参见 ZYMO RESEARCH 生物公司 EZ DNA Methylation-Direct™试剂盒说明书[16]。

1.3.2　甲基化的 PCR 检测　甲基化人 SHOX2 和 RASSF1A 基因检测试剂盒、引物和探针均由上海透景生命科技股份有限公司提供。PCR 反应液包含 3 μL 扩增引物,1.5 μL 标记探针, 4.8 μL 2×Taq 缓冲液(包括 dNTP 和 Taq 聚合酶)和 10.7 μL ddH_2O;质控品为测序确认的 SHOX2 和 RASSFlA 甲基化阳性 DNA;阴性对照为纯化水。向 PCR 反应管中每孔加入上述混匀的 PCR 反应液 20 μL,再加入样本 DNA 5 μL,预留两管加入质控品、阴性对照各 5 μL。PCR 反应条件:95 ℃预变性 10 min;95 ℃变性 15 s,60 ℃退火 30 s(共 5 个循环);95 ℃变性 15 s,57 ℃退火 30 s(共 40 个循环)。在最后循环阶段 60 ℃时收集 FAM、VIC 及 CY5 的信号。

1.3.3　甲基化的 Sanger 测序法检测　使用 Primer Premier 5.0 设计引物,SHOX2 正向引物序列:5'-GGTGTTGTGTCGTATAGGGAGT-3',反向引物序列:5'-TCCGCCTCCTACCTTCTAAC-3';RASSF1A 正向引物序列:5'-GAGGGAAGGAAGGGTAAGG-3',反向引物序列:5'-GAGGGAAGGAAGGGTAAGG-3'。PCR 反应体系:共 40 μL,包含 5 μL cDNA,10 μmol/L 正、反向引物各 0.8 μL,20 μL 2×Taq 缓冲液(包括 dNTP 和 Taq 聚合酶)和 13.4 μL ddH_2O。PCR 反应条件:95 ℃预变性 10 min,95 ℃变性 30 s(共 45 个循环),58 ℃退火 35 s,72 ℃延伸 30 s,最后 72 ℃延伸 8 min。产物由生工生物工程(上海)股份有限公司进行 Sanger 测序。

1.4　随访　对病理学诊断阴性而 SHOX2、RASSF1A 基因甲基化PCR法检测阳性的28例患者进行门诊或电话随访,随访时间为 6 个月,终点事件为疾病恶化。

1.5　统计学处理　采用 SPSS 22.0 进行数据分析。对 PCR 和 Sanger 测序法检测结果进行 *Kappa* 一致性检验,应用χ^2 检验比较肺癌组和肺部良性疾病组 BALF 中 SHOX2 和 RASSF1A 基因甲基化阳性率的差异,应用 ROC 曲线评价单一指标和多个指标联合检测的价值,并计算 *AUC* 及 95% *CI*;检验水准 $\alpha=0.05$。

2　结果

2.1　PCR 和 Sanger 测序法检测结果的一致性分析　见表 1。结果显示,两种方法在检测 SHOX2 和 RASSF1A 基因甲基化上有较好的一致性,*Kappa* = 0.978, 95% *CI* = 0.954 ~ 1.000。

表 1　PCR 和 Sanger 测序法一致性比较

PCR	Sanger 测序法		总计
	阳性	阴性	
阳性	138	3	141
阴性	0	135	135
总计	138	138	276

2.2　肺癌组、良性疾病组 BALF 中 SHOX2 和 RASSF1A 基因甲基化 PCR 检测结果　见表 2。按病理类型分层,SHOX2、RASSF1A 两基因甲基化 PCR 检测同时阳性者在小细胞肺癌患者中最高,为 93.5%,而肺腺癌和肺鳞癌较低,仅为 40.4% 和 36.4%;但两基因甲基化 PCR 联合检测时,任一阳性者肺腺癌、鳞癌检出率分别达到 66.0% 和 90.9%(表 3)。不同 TNM 分期肺癌患者 BALF 中 SHOX2 和 RASSF1A 基因甲基化联合检测阳性率均高于单一指标检测阳性率;晚期肺癌患者 BALF 中基因甲基化的阳性率最高,为 89.3%(表 3)。

表 2　肺癌组、良性疾病组 BALF 中 SHOX2 和 RASSF1A 基因甲基化 PCR 检测结果　例(%)

组别	*n*	SHOX2	RASSF1A	二者均阳性	任一阳性
肺癌组	131	101(77.1)	77(58.8)	68(51.9)	110(84.0)
良性疾病组	145	22(15.2)	12(8.3)	6(4.1)	28(19.3)
χ^2		106.834	80.343	80.036	115.093
P		<0.001	<0.001	<0.001	<0.001

万方数据

表3 不同病理类型及疾病分期肺癌患者 BALF 中基因甲基化 PCR 检测结果 例(%)

项目	n	SHOX2	RASSF1A	二者均阳性	任一阳性
肺癌病理类型					
肺腺癌	47	27(57.4)	23(48.9)	19(40.4)	31(66.0)
肺鳞癌	44	37(84.1)	19(43.2)	16(36.4)	40(90.9)
小细胞肺癌	31	30(96.8)	30(96.8)	29(93.5)	31(100.0)
大细胞肺癌	5	5(100.0)	3(60.0)	3(60.0)	5(100.0)
其他类型	4	2(50.0)	2(50.0)	1(25.0)	3(75.0)
肺癌分期					
Stage 0 期	5	1(20.0)	2(40.0)	0(0)	3(60.0)
Stage Ⅰ期	18	13(72.2)	8(44.4)	8(44.4)	13(72.2)
Stage Ⅱ期	32	26(81.2)	19(59.3)	17(53.1)	28(87.5)
Stage Ⅲ期	39	31(79.5)	25(64.1)	22(56.4))	34(87.2)
Stage Ⅳ期	28	23(82.1)	19(67.9)	17(60.7)	25(89.3)
分期未明	9	7(77.8)	4(44.4)	4(44.4)	7(77.8)

2.3 BALF 细胞学联合基因甲基化 PCR 检测的价值比较

见表4。

表4 单一指标与多个指标联合检测结果

指标	灵敏度	特异度	约登指数	*AUC* 及 95% *CI*
BALF 细胞学	0.435	1.000	0.435	0.782(0.725~0.840)
RASSF1A	0.588	0.891	0.473	0.762(0.703~0.826)
SHOX2	0.771	0.848	0.619	0.810(0.756~0.863)
SHOX2+RASSF1A*	0.840	0.807	0.647	0.823(0.771~0.875)
细胞学+SHOX2*	0.893	0.848	0.741	0.871(0.825~0.916)
细胞学+RASSF1A*	0.832	0.917	0.749	0.875(0.829~0.920)
细胞学+SHOX2+RASSF1A*	0.931	0.807	0.738	0.869(0.823~0.915)

*:各指标间并联

2.4 良性疾病组中 SHOX2 和 RASSF1A 基因甲基化阳性患者的随访研究

在 145 例肺良性疾病患者中,有 28 例 SHOX2 和(或)RASSF1A 基因甲基化检测阳性,临床诊断为肺炎、支气管扩张、呼吸道淀粉样变、肺结核、结节病等。对上述 28 例患者进行随访研究,6 个月随访期间有 2 例病情恶化,后经病理确诊为肺癌。

3 讨论

DNA 甲基化已广泛用于癌症的辅助诊断,尤其适合微创获取标本(如 BALF、胸腔积液等)的检测[17-18]。多项研究[9,13,19]显示,SHOX2 和 RASSF1A 基因异常甲基化对肺癌具有诊断价值,可作为肺癌的生物标志物。本研究中作者使用 PCR 和 Sanger 测序法对 276 名疑似肺癌患者 BALF 标本中 SHOX2 和 RASSF1A 基因甲基化进行检测,结果显示两种方法检测结果一致性良好,说明 PCR 法在 DNA 甲基化分析中是可靠的,而且检测成本更低,对样本 DNA 含量的要求低于 Sanger 测序法,这些优势是其广泛推广应用的前提。本研究中,SHOX2 和 RASSF1A 基因甲基化联合检测对肺癌的诊断灵敏度为 0.840,特异度为 0.807,表明 SHOX2 和 RASSF1A 甲基化联合检测具有区分恶性和良性肺部疾病的诊断效能。

目前,病理学仍然是肺癌诊断金标准;但临床中受病灶组织异质性、病灶部位等因素影响,病理学检查有时呈现阴性结果,导致诊断困难,因此迫切需要多种检测方法相结合以提高肺癌的诊断率。Darwiche 等[20]使用组织学与 SHOX2 基因甲基化测定相结合对肺癌淋巴结样本进行检测,达到 99% 的检测灵敏度和 99% 的特异性。在本研究中,肺癌组 BALF 细胞学诊断灵敏度仅为 0.435;与单一指标检测相比,当 SHOX2、RASSF1A 两指标联合或者基因甲基化与细胞学两两联合时,诊断灵敏度显著升高。当 SHOX2、RASSF1A 基因甲基化和细胞学三指标联合使用时进一步将诊断灵敏度提高至 0.931,这表明 BALF 中 SHOX2 和 RASSF1A 甲基化检测可以作为肺癌细胞学诊断的有效补充工具。

有研究[10]显示,SHOX2、RASSF1A 基因甲基化阳性检出率与肺癌病理类型之间存在相关性。本研究中,SHOX2、RASSF1A 基因甲基化联合检测阳性率在小细胞肺癌和肺鳞癌中分别是 100% 和 90.9%,肺腺癌组最低(大细胞肺癌例数过少,仅 5 例,其阳性检测率不能提供可靠信息),表明 SHOX2、RASSF1A 基因甲基化检测对小细胞肺癌和肺鳞癌的敏感性高于肺腺癌,这与以前的研究结论相似[12,15];分析原因可能与小细胞肺癌和肺鳞癌中存在高水平 SHOX2 基因甲基化有关。本研究结果还显示,SHOX2、RASSF1A 基因甲基化水平与肺癌病情分期之间亦有相关性,晚期肺癌患者 SHOX2、RASSF1A 基因甲基化水平高于早期肺癌(89.3% *vs* 72.2%);这与晚期肺癌更具侵袭性、肿瘤生长更快、肿瘤性坏死更多,进而导致 DNA 释放增多,更容易检测到甲基化等因素有关[14]。

本研究中,肺良性疾病组有 28 例患者 BALF 中 SHOX2、RASSF1A 基因甲基化检测阳性,在随访过程中 2 例随后被确诊为肺癌;提示对可疑患者进行 DNA 甲基化检测是必要的,尤其对病理学检测结果阴性而甲基化分析阳性患者,至少可以提前预警,密切随访,必要时可多次穿刺活检以免漏、误诊。

总之,BALF 中 SHOX2、RASSF1A 基因甲基化

万方数据

分析具有区分恶性和良性肺部疾病的诊断效能;SHOX2、RASSF1A 基因甲基化分析与细胞学联合使用可显著提高肺癌诊断灵敏度,是肺癌病理学诊断的有效补充工具。

参考文献

[1] ZENG H,CHEN W,ZHENG R,et al. Changing cancer survival in China during 2003-15: a pooled analysis of 17 population-based cancer registries[J]. Lancet Glob Health,2018,6(5):e555

[2] ETTINGER DS,WOOD DE,AISNER DL,et al. Non-small cell lung cancer,Version 5. 2017,NCCN clinical practice guidelines in oncology[J]. J Natl Compr Canc Netw,2017,15(4):504

[3] LIANG MZ,TANG W,XU DM,et al. Low-dose CT screening for lung cancer: computer-aided detection of missed lung cancers[J]. Radiology,2016,281(1):279

[4] OST DE,ERNST A,LEI X,et al. Diagnostic yield and complications of bronchoscopy for peripheral lung lesions: results of the AQuIRE registry[J]. Am J Respir Crit Care Med,2016,193(1):68

[5] DOR Y,CEDAR H. Principles of DNA methylation and their implications for biology and medicine[J]. Lancet,2018,392(1149):777

[6] SHEAFFER KL,ELLIOTT EN,KAESTNER KH. DNA hypomethylation contributes to genomic instability and intestinal cancer initiation[J]. Cancer Prev Res(Phila),2016,9(7):534

[7] 刘莹,王圆圆,贾文青,等. 肺癌患者血清 p16、FHIT、APC 基因甲基化检测[J]. 郑州大学学报(医学版),2017,52(1):51

[8] 王威,冯晓蕾,段晓冉,等. 基于 3 种基因启动子甲基化联合端粒长度构建肺癌诊断支持向量机模型[J]. 郑州大学学报(医学版),2015,50(4):462

[9] WEISS G,SCHLEGEL A,KOTTWITZ D,et al. Validation of the SHOX2/PTGER4 DNA methylation marker panel for plasma-based discrimination between patients with malignant and nonmalignant lung disease[J]. J Thorac Oncol,2017,12(1):77

[10] SONG LL,YU HT,LI YE. Diagnosis of lung cancer by SHOX2 gene methylation assay[J]. Mol Diagn Ther,2015,19(3):159

[11] Ilse P,Biesterfeld S,Pomjanski N,et al. Analysis of SHOX2 methylation as an aid to cytology in lung cancer diagnosis[J]. Cancer Genomics Proteomics,2014,11(5):251

[12] MARI-ALEXANDRE J,DIAZ-LAGARES A,VILLALBA M,et al. Translating cancer epigenomics into the clinic:focus on lung cancer[J]. Transl Res,2017,189:76

[13] DUBOIS F,KELLER M,CALVAYRAC O,et al. RASSF1A suppresses the invasion and metastatic potential of human non-small cell lung cancer cells by inhibiting YAP activation through the GEF-H1/RhoB pathway[J]. Cancer Res,2016,76(6):1627

[14] GAO L,XIE EF,YU TF,et al. Methylated APC and RASSF1A in multiple specimens contribute to the differential diagnosis of patients with undetermined solitary pulmonary nodules[J]. J Thorac Dis,2015,7(3):422

[15] DU RAND IA,BLAIKLEY J,BOOTON R,et al. Summary of the British Thoracic Society guideline for diagnostic flexible bronchoscopy in adults[J],Thorax,2013,68(8):786

[16] REN MP,WANG CH,SHENG DL,et al. Methylation analysis of SHOX2 and RASSF1A in bronchoalveolar lavage fluid for early lung cancer diagnosis[J]. Ann Diagn Pathol,2017,27:57

[17] KOCH A,JOOSTEN SC,FENG Z,et al. Analysis of DNA methylation in cancer: location revisited[J]. Nat Rev Clin Oncol,2018,15(7):459

[18] LIU L,TOUNG JM,JASSOWICZ AF,et al. Targeted methylation sequencing of plasma cell-free DNA for cancer detection and classification[J]. Ann Oncol,2018,29(6):1445

[19] 魏慧君,房念珍,郭丽丽,等. RASSF1A 基因启动子甲基化与非小细胞肺癌关系的 meta 分析[J]. 中国肺癌杂志,2015,18(7):443

[20] DARWICHE K,Zarogoulidis P,Baehner K,et al. Assessment of SHOX2 methylation in EBUS-TBNA specimen improves accuracy in lung cancer staging[J]. Ann Oncol,2013,24(11):2866

(2019-03-01 收稿　责任编辑赵秋民)

万方数据
