# Supplementary material for: Meta-analysis of the accuracy for RASSF1A methylation in bronchial aspirates for the diagnosis of lung cancer
Source: PLoS One. 2024 Jul 25;19(7):e0299447. doi: 10.1371/journal.pone.0299447 (PMC11271935; doi:10.1371/journal.pone.0299447)
Supplement: S1 File — (ZIP) [file pone.0299447.s006.zip › S1 File/Liu JJ 2021.pdf]

高分辨 CT 三维重建联合肺泡灌洗液中 SHOX2 和 RASSF1A 基因甲基化检测诊断早期肺结节的效能分析

刘家杰　肖泽林　庄仕龙

【摘要】 目的　分析高分辨 CT 三维重建联合肺泡灌洗液中矮小同源盒基因 2(SHOX2) 和 Ras 相关区域家族 1A(RASSF1A) 基因甲基化检测在早期肺结节中的诊断效能。方法　160 例肺结节患者为研究对象，后期经 CT 引导下经皮肺部病理穿刺活检确诊小细胞肺癌 (肺恶性肿瘤直径≤ 3 cm) 患者 60 例，良性肺结节患者 100 例。所有患者先后进行高分辨 CT 联合肺泡灌洗液细胞学检测，肺泡灌洗液中 SHOX2 和 RASSF1A 基因甲基化检测，高分辨 CT 三维重建联合肺泡灌洗液中 SHOX2 和 RASSF1A 基因甲基化检测。以 CT 引导下经皮肺部病理穿刺活检为金标准，对比三种检测方式诊断早期肺结节的效能。结果　高分辨 CT 三维重建联合肺泡灌洗液中 SHOX2 和 RASSF1A 基因甲基化检测的灵敏度 96.67%、准确率 96.25% 高于高分辨 CT 联合肺泡灌洗液细胞学检测 (灵敏度 83.33%、准确率 84.38%)、肺泡灌洗液中 SHOX2 和 RASSF1A 基因甲基化检测 (灵敏度 86.67%、准确率 88.75%), 高分辨 CT 三维重建联合肺泡灌洗液中 SHOX2 和 RASSF1A 基因甲基化检测的特异度 96.00% 高于高分辨 CT 联合肺泡灌洗液细胞学检测的 85.00%, 差异具有统计学意义 (P<0.05)。高分辨 CT 联合肺泡灌洗液细胞学检测、肺泡灌洗液中 SHOX2 和 RASSF1A 基因甲基化检测的灵敏度、准确率比较，差异无统计学意义 (P>0.05)；肺泡灌洗液中 SHOX2 和 RASSF1A 基因甲基化检测、高分辨 CT 三维重建联合肺泡灌洗液中 SHOX2 和 RASSF1A 基因甲基化检测的特异度比较，差异无统计学意义 (P>0.05)。结论　高分辨 CT 三维重建联合肺泡灌洗液中 SHOX2 和 RASSF1A 基因甲基化检测方式显著优于单一指标检测与高分辨 CT 联合肺泡灌洗液细胞学检测。

【关键词】 高分辨 CT；三维重建；肺泡灌洗液；矮小同源盒基因 2；Ras 相关区域家族 1A；联合检测；诊断效能

DOI：10.14163/j.cnki.11-5547/r.2021.17.034

目前肺癌发病率与病死率占全球恶性肿瘤发病率和病死率的第 1 位，而且随着人们生活水平的提高，肺癌患者年龄日趋年轻化，因此该疾病已成为广大医生及患者较为重视的问题。通常可分为非小细胞肺癌和小细胞肺癌 2 种类型，但由于肺泡没有感觉神经，无法感受到疼痛，因此早期肺癌没有较典型的临床症状，多数患者难以及时确诊，当出现咳嗽咳痰、咳血、胸痛时可能已发展到晚期[1]。肺结节作为肺癌早期主要临床表现之一，一般可通过穿刺病理活检进行确诊，但对于较小的肺结节，其诊断率会受到实质成分、结节结构、技术要求等因素的影响。据相关数据显示，高分辨 CT 三维重建联合肺泡灌洗液中 SHOX2 和 RASSF1A 基因甲基化检测在诊断早期肺结节与早期肺癌中的应用价值较好，尤其是较小的单发性肺结节的检出率较高，能减少早期肺癌的漏诊及误诊率[2,3]。基于此，本研究将本院 60 例小细胞肺癌患者及 100 例良性肺结节患者作为观察对象，进一步分析高分辨 CT 三维重建联合肺泡灌洗液中 SHOX2 和 RASSF1A 基因甲基化检测的诊断优势，以期为早期肺癌临床诊断提供科学的理论依据与实践依据。

1　资料与方法

1. 1　一般资料　选取本院 2019 年 1 月 ~2020 年 12 月收治的 160 例肺结节患者为研究对象，后期经 CT 引导下经皮肺部病理穿刺活检确诊的小细胞肺癌 (肺恶性肿瘤直径≤ 3 cm) 患者 60 例，良性肺结节患者 100 例。小细胞肺癌患者男 43 例，女 17 例；年龄 30~65 岁，平均年龄 (52.76 ± 11.53) 岁。良性肺结节患者男 69 例，女 31 例；年龄 25~68 岁，平均年龄 (51.89 ± 12.68) 岁。两种疾病患者的一般资料比较，差异无统计学意义 (P>0.05), 具有可比性。

1. 2　纳入及排除标准

1. 2. 1　纳入标准　①均符合肺结节相关诊断标准[4]，且临床资料完整详实；②视觉、听觉等功能正常；③患者及其家属对本研究知情同意。

1. 2. 2　排除标准　①非肺部肿瘤患者；②严重传染病患者；③依从性较差的患者；④严重精神障碍患者。

1. 3　方法　所有患者先后进行高分辨 CT 联合肺泡灌洗液细胞学检测，肺泡灌洗液中 SHOX2 和 RASSF1A

基金项目：广东省医学科学技术研究基金项目 (项目编号：B20211343)
作者单位：510060　广州市胸科医院外一科

万方数据

基因甲基化检测，高分辨 CT 三维重建联合肺泡灌洗液中 SHOX2 和 RASSF1A 基因甲基化检测。

1. 3. 1　高分辨 CT 联合肺泡灌洗液细胞学检测　高分辨螺旋 CT 常规胸部扫描，参数分别为层距 10 mm、层厚 10 mm、矩阵 512×512、螺距 1.0~1.1 mmm、时间 1 s、电流 140 mA、电压 120 kV，在标准图像重建算法的基础上对患者病灶区域进行高分辨 CT 扫描，设置层距 1.0~3.0 mm，层厚 1.0~1.5 mm，扫描视野为 24~35 cm，高分辨精细算法条件为电流 170 mA、电压 140 kV；然后于局部麻醉状态下根据影像学定位将支气管镜放置于患者病灶部位，应用生理盐水反复灌洗，回收灌洗液 50 ml，除去杂质与黏液后 1500 r/min 离心 10 min，应用 BP-MI1640 培养液与 10% 小牛血清培养液于 37℃培养箱中培养 1 h 后采集细胞，并应用抗 $CD3^+$、抗 $CD4^+$、抗 $CD8^+$、单克隆抗体染色后对 T 淋巴细胞亚群进行检测。

1. 3. 2　肺泡灌洗液中 SHOX2 和 RASSF1A 基因甲基化检测　取患者肺泡灌洗液 10 ml，行 10000 r/min 离心 5 min，除去上清液，将剩余沉淀用于提取 DNA，提取 DNA 结束后应用亚硫酸盐进行修饰；应用 Primer 5.0 设计引物，SHOX2 的正向引物序列为：5'-GGTGTTGTGTCGTATAGGGAGT-3'，反向引物序列为：5'-TCCGCCTCCTACCTTCTAAC-3'；RASSF1A 的正向引物序列为：5'-GAGGGAAGGAAGGGTAAGG-3'，反向引物序列为：5'-GAGGGAAGGAAGGGTAAGG-3'。测序聚合酶链式反应 (PCR) 体系 (40 μl)：将修饰后的 DNA 5 μl，上游引物 (10 μmol/L)0.8 μl，下游引物 (10 μmol/L)0.8 μl，2×Taq 缓冲液（含有 Taq 酶与 dNTPs）加水 13.4 μl；PCR 扩增：PCR 预变性 95℃ 10 min，循环 95 ℃ 30 s，58 ℃ 35 s，72 ℃ 30 s，45 个循环。延伸 72℃ 8 min，上述 PCR 产物经检测合格后送生物工程有限公司测序。

1. 3. 3　高分辨 CT 三维重建　行高分辨 CT 常规扫描后，将二维图像传入工作站，应用 Hitachi 3D Display 软件进行三维容积处理：确定上阈值为 -250~350 Hu 和下阈值为 -650~800 Hu，划出兴趣区，选择参与重建的断层图像（参与重建的断层图像应当是同一扫描序列的连续层面，包括患者病灶每一个病变的层面与上下 2~4 层），三维图像显示只有根据具体需要行进一步调整，最大程度地显示病灶处病变状况及病灶周围支气管与肺血管状况，三维图像显示之后对图像的亮度和对比度进行适当调节，使其达到最佳的视觉效果。同时对病灶进行旋转、切割，进一步观察病灶内部的边缘、结构以及相邻的血管及支气管的状况。

1. 4　观察指标　以 CT 引导下经皮肺部病理穿刺活检为金标准，对比三种检测方式诊断早期肺结节的效能。

1. 5　统计学方法　采用 SPSS21.0 统计学软件进行统计分析。计量资料以均数 ± 标准差 ($\bar{x}\pm s$) 表示，采用 t 检验；计数资料以率 (%) 表示，采用 χ^2 检验。$P<0.05$ 表示差异具有统计学意义。

2　结果

高分辨 CT 三维重建联合肺泡灌洗液中 SHOX2 和 RASSF1A 基因甲基化检测的灵敏度、准确率高于高分辨 CT 联合肺泡灌洗液细胞学检测、肺泡灌洗液中 SHOX2 和 RASSF1A 基因甲基化检测，高分辨 CT 三维重建联合肺泡灌洗液中 SHOX2 和 RASSF1A 基因甲基化检测的特异度高于高分辨 CT 联合肺泡灌洗液细胞学检测，差异具有统计学意义 ($P<0.05$)。高分辨 CT 联合肺泡灌洗液细胞学检测、肺泡灌洗液中 SHOX2 和 RASSF1A 基因甲基化检测的灵敏度、准确率比较，差异无统计学意义 ($P>0.05$)；肺泡灌洗液中 SHOX2 和 RASSF1A 基因甲基化检测、高分辨 CT 三维重建联合肺泡灌洗液中 SHOX2 和 RASSF1A 基因甲基化检测的特异度比较，差异无统计学意义 ($P>0.05$)。见表 1，表 2。

表 1　不同检测方式的诊断结果 (n)

检测方式		病理诊断		合计
		恶性	良性	
高分辨 CT 联合肺泡灌洗液细胞学检测	恶性	50	15	65
	良性	10	85	95
肺泡灌洗液中 SHOX2 和 RASSF1A 基因甲基化检测	恶性	52	10	62
	良性	8	90	98
高分辨 CT 三维重建联合肺泡灌洗液中 SHOX2 和 RASSF1A 基因甲基化检测	恶性	58	4	62
	良性	2	96	98
合计		60	100	160

表 2　不同检测方式的灵敏度、特异度及准确率比较 (%)

检测方式	灵敏度	特异度	准确率
高分辨 CT 联合肺泡灌洗液细胞学检测	83.33(50/60)[a]	85.00(85/100)[a]	84.38(135/160)[a]
肺泡灌洗液中 SHOX2 和 RASSF1A 基因甲基化检测	86.67(52/60)[a]	90.00(90/100)	88.75(142/160)[a]
高分辨 CT 三维重建联合肺泡灌洗液中 SHOX2 和 RASSF1A 基因甲基化检测	96.67(58/60)	96.00(96/100)	96.25(154/160)

注：与高分辨 CT 三维重建联合肺泡灌洗液中 SHOX2 和 RASSF1A 基因甲基化检测比较，[a]$P<0.05$

万方数据

3 讨论

据临床数据显示，肺癌患者 5 年存活率 <12.00%，若早期及时给予准确诊断及治疗措施，其存活率可提升至 70.00%，因此近几年肺小结节与早期肺癌的诊断备受临床医师关注，随着我国医学影像技术的不断发展以及 CT 在临床的广泛应用，加上高分辨 CT 三维重建技术能更细腻地显示出肺结节征象，从多方位角度对患者病变部位进行形态学分析，从而使得肺结节的检出率愈来愈高，即便如此 CT 检测在早期肺癌诊断中依然存在某些不足之处，就算是高分辨 CT 在图像显示中也仅见纵隔创伤病灶结节中的实性成分，在一定程度上会直接影响肺结节诊断准确率[5]。此外，林列坤等[6]的研究结果显示，等电子纤维支气管镜取样后的肺泡灌洗液进行基因甲基化检测能够减少恶性肿瘤患者的机体损伤，不仅能改善肺癌患者早期诊断准确率，还能进一步提高其生存率。但迄今为止，鲜有高分辨 CT 三维重建联合肺泡灌洗液中 SHOX2 和 RASSF1A 基因甲基化检测诊断分辨早期肺结节与早期肺癌相关的研究，因此本研究将高分辨 CT 联合肺泡灌洗液细胞学检测与高分辨 CT 三维重建联合肺泡灌洗液中 SHOX2 和 RASSF1A 基因甲基化检测用于早期肺结节诊断中。

本研究诊断结果显示，高分辨 CT 三维重建联合肺泡灌洗液中 SHOX2 和 RASSF1A 基因甲基化检测的灵敏度 96.67%、准确率 96.25% 高于高分辨 CT 联合肺泡灌洗液细胞学检测 (灵敏度 83.33%、准确率 84.38%)、肺泡灌洗液中 SHOX2 和 RASSF1A 基因甲基化检测 (灵敏度 86.67%、准确率 88.75%)，高分辨 CT 三维重建联合肺泡灌洗液中 SHOX2 和 RASSF1A 基因甲基化检测的特异度 96.00% 高于高分辨 CT 联合肺泡灌洗液细胞学检测的 85.00%，差异具有统计学意义 ($P<0.05$)。表明高分辨 CT 三维重建联合肺泡灌洗液中 SHOX2 和 RASSF1A 基因甲基化检测方式在早期肺结节中的诊断效能更高，分析原因可能是首先通过多方位多层面的高分辨 CT 三维重建技术显示肺部结节征象，清楚地显示支气管、胸膜、血管等与肺部结节的关系，以便于早期恶性结节的诊断[7]；而肺泡灌洗液检测是指通过微生物学及化学方法对肺泡灌洗液进行分析，利用纤维支气管镜对支气管以下肺段和亚肺段进行灌洗后，采集肺泡表面衬液可获得肺泡灌洗液，对其进行实验室检查；而 DNA 甲基化分子标志物 SHOX2 对小细胞肺癌和肺鳞癌的诊断敏感性较高；RASSF1A 在肿瘤发生发展过程中有着重要的作用，在肺癌中有着较高的表达，尤其与肺腺癌的发生有着密切的关联性，因此 SHOX2 和 RASSF1A 基因亦可作为早期肺结节鉴别条件之一，这与田祺等[8]的研究结果具有一致性。

综上所述，高分辨 CT 三维重建联合肺泡灌洗液中 SHOX2 和 RASSF1A 基因甲基化检测用于早期肺结节诊断中，可有效提高其准确率，以减少误诊率和漏诊率，值得推广。

参 考 文 献

[1] 赖红锦，林锋，陈楠，等．肺癌干细胞作为靶点的肺癌治疗策略研究进展．中国肺癌杂志，2018, 21(1):57-62.

[2] 张毅敏，王明丽，吴杰，等．肺泡灌洗液中 SHOX2 和 RASSF1A 基因甲基化联合检测对肺癌的诊断价值．肿瘤学杂志 2016, 22(12):1032-1035.

[3] 宋乐乐，李月敏．SHOX2 基因甲基化辅助诊断肺癌的临床转化状况．中国肿瘤生物治疗杂志，2016, 23(4):550-554.

[4] 张碧云，陈自谦，赵政文，等．计算机辅助检测系统对数字化胸片中肺结节诊断的应用价值．中华放射学杂志，2005, 39(10):1092.

[5] 张振显，杨爱莲，吴爱军，等．多层螺旋 CT 动脉扫描及三维重建在孤立性肺小结节诊断中的应用比较．中国医学装备，2018, 15(9):62-65.

[6] 林列坤，卢春生，郑义，等．支气管肺泡灌洗液 SHOX2 和 RASSF1A 基因甲基化检测在临床肺癌诊断中的应用．中国实用医药，2019, 14(18):1-3.

[7] 范明新，步玉兰，张学成，等．高分辨率 CT 对纯磨玻璃密度结节良恶性诊断价值探讨．中华肿瘤防治杂志，2018, 25(15): 1094-1096.

[8] 田祺，赵靖，刘菲菲，等．支气管肺泡灌洗液 SHOX2 和 RASSF1A 基因甲基化检测在临床肺癌早期诊断中的应用．中国老年学杂志，2020, 40(9):3629-3632.

［收稿日期：2021-01-27］

万方数据
