# Supplementary material for: Meta-analysis of the accuracy for RASSF1A methylation in bronchial aspirates for the diagnosis of lung cancer
Source: PLoS One. 2024 Jul 25;19(7):e0299447. doi: 10.1371/journal.pone.0299447 (PMC11271935; doi:10.1371/journal.pone.0299447)
Supplement: S1 File — (ZIP) [file pone.0299447.s006.zip › S1 File/Yu ZT 2007.pdf]

·论著·

肺癌患者 Ras相关区域家族 1A基因启动子异常甲基化的检测

余宗涛 袁亚莉 张吉才 高琼 吕军

[中图分类号] R734.2 [文献标识码] A [文章编号] 1001-9057(2007)01-0023-03

[摘要] **目的** 探讨肺癌组织和外周血浆、支气管肺泡灌洗液(BALF)中 Ras相关区域家族 1A(RASSF1A)基因启动子异常甲基化状况及其在肺癌诊断中的价值。**方法** 用甲基化特异 PCR方法对肺癌患者癌组织、癌旁组织及相应血浆、BALF进行 RASSF1A异常甲基化检测。**结果** 45例肺癌组织中,RASSF1A基因启动子异常甲基化率为 53.33% (24/45),相应血浆中 RASSF1A的甲基化检出率为 28.89% (13/45),BALF检出率为 42.22% (19/45),而癌旁组织中的 RASSF1A启动子甲基化检出率为 13.04% (3/23)、正常对照血浆、非肺癌患者 BALF中未检出甲基化,只检出未甲基化的 RASSF1A。血浆、BALF中甲基化改变与肿瘤组织甲基化状况显著相关($P<0.01$);但与患者年龄、性别、肿瘤大小、恶性程度、肿瘤分类的差异无统计学意义($P>0.05$)。**结论** 血浆、BALF中 RASSF1A基因异常甲基化改变的检测在肺癌的特异诊断等方面有一定的应用价值。

[关键词] 肺肿瘤; DNA甲基化; RASSF1A基因; 血浆; PCR; BALF

Detection of RASSF1A gene promoter hypermethylation in patients with lung cancer

YU Zong-tao, YUAN Ya-li, ZHANG Ji-cai, et al. Department of Clinical laboratory, Taihe Hospital, Affiliated Yunyang Medicinal College, Shiyan 442000, China

[Abstract] Objective To investigate the status and diagnostic value of hypermethylated RASSF1A gene promoter in cancer tissue and peripheral plasma and BALF of patients with lung cancer. Methods We analyzed the hypermethylation status of RASSF1A gene in 45 lung cancer tissues, 23 para-cancer tissues and corresponding blood plasma and BALF by methylation specific-PCR. Results The frequency of methylation of Promoter of RASSF1A gene was 53.33% (24/45) in lung cancer, 28.89% (13/45) in peripheral blood plasma, 42.22% (19/45) in corresponding BALF, 13.04% (3/23) in lung carcinoma adjacent tissues, respectively. No methylation had being present in normal plasma and BALF controls ($P<0.01$), which were significantly coincident and higher in tumors than para-tumor, but it was not correlated with clinical data, such as sex, age, pathological stage, pathological type and tumor size ($P>0.05$). Conclusion Detection of aberrant methylation change of RASSF1A promoter in plasma and/or BALF may contribute to a definite value in early prognostic in lung cancer.

[Key words] Lung cancer; DNA methylation; RASSF1SA gene; Plasma; BALF; PCR

肺癌是当今世界各国常见的恶性肿瘤,其发病率和死亡率均占第一位,提高其早期期诊断率是其治疗的关键,应用分子生物学的知识和技术对支气管肺泡灌洗液(BALF)和血液中肿瘤相关基因异常的检测成为高危人群的肺癌筛选手段。肺癌的发生与抑癌基因的失活密切相关,目前随着表观遗传学研究的发展,抑癌基因启动子区 CpG岛的高甲基化已被当作引起转录沉默的普遍机制。Ras相关区域家族 1A(RASSF1A)是 2000年从 3号染色体短臂(3p21.3)上克隆出来的新型候选抑癌基因,本研究 RASSF1A在肺癌组织及其相应血浆、BALF和肺癌旁组织启动子甲基化状态,分析启动子甲基化和肺癌早期诊断和预后评估等的临床关系。

基金项目:湖南省衡阳科技局资助项目(06KS13)

作者单位:442000湖北十堰,郧阳医学院附属太和医院检验科(余宗涛、张吉才、高琼、吕军);湖南 衡阳南华大学生命科学与技术学院(袁亚莉)

材料与方法

1. 材料

(1)标本来源:收集湖北省十堰市太和医院 2005年 3月～2006年 2月手术切除肺癌组织 45例,男 28例,女 17例。年龄 45～78岁,平均年龄 55.3岁。其中高分化 23例、中分化 10例、低分化 12例;小细胞肺癌(SLLC)8例、非小细胞肺癌(NSCLC)37例;临床TNM分期:Ⅰ级 25例,Ⅱ级 18例,Ⅲa级 2例(由于Ⅲ

(C)1994-2022 China Academic Journal Electronic Publishing House. All rights reserved. http://www.cnki.net

b、Ⅳ级失去手术期，固无收集）。全部标本均经病理证实，取上述45例肺癌切除病人距离肿瘤边缘2 cm外组织作为对照组织23例（注：另22例由于肿瘤边缘不清没有切取）；同时抽此肺癌和非肺癌患者（包括结核、肺炎病人）各45例抗凝血（EDTA-K2）5 ml立即离心，取血浆层，－80℃冰箱保存备用。肺癌患者及非肺癌患者各45例BALF的获得：将支气管镜前端嵌入右肺中叶的段或亚段支气管中，灌注生理盐水（1 ml/kg）后立即回收BALF，反复3次，离心后分离细胞成分备用。

（2）主要试剂与仪器：Wizard DNA clean up纯化试剂盒（美国Promega公司）；Taq DNA聚合酶、dNTP、10×Buffer、糖原、蛋白酶K（华美公司）；SYBR Green Ⅰ（基因公司）；对苯二酚和亚硫酸氢钠（美国Sigma公司），GeneRuleTM 50 bp DNA marker、GeneRuleTM 100 bp DNA marker（MBI）；PE7000（美国ABI公司）、DU640紫外分光光度计（德国贝克曼公司）、ZF型紫外分析仪（上海康华）、低温离心机（日本Hitachi）。

（3）引物合成：RASSF1A基因甲基化引物[1]（5'-GTGTTAACGCGTTGCGTATC-3'；5'-AACCCCGCGAACTAAAAACGA-3'）扩增片段长度为93bp，RASSF1A基因非甲基化引物[2]（5'-TTTGGTTGGAGTGTGTTAATGTG-3'；5'-CAAACCCCACAAACTAAAAACAA-3'）扩增片段长度为105bp（北京赛百盛公司）。

2. 方法

（1）组织、细胞、血浆DNA提取：以经典酚氯仿抽提法提取组织DNA，经紫外260 nm定量后于－80℃保存备用。

（2）基因组DNA的磺化修饰：参照文献[3]，取4 μg（总体积为50 μl）基因组DNA，使其变性、碱基修饰、脱盐回收再脱去磺化基团后，用预冷酒精沉淀回收。离心干燥后重新溶于50 μl去离子水中，置－40℃备用。

（3）甲基化特异性聚合酶链反应（Methylation-Specific PCR，MS-PCR）：PCR反应体系为30 μl包括磺化修饰后的基因组DNA 50～100 ng（3 μl），10×buffer 3 μl，2 mmol/L dNTP 3 μl，25 mmol/L MgCl2 3 μl，Taq酶2 U（1 μl），上下游引物各2 μl（12 pmol），20 XSYBR GreenⅠ 0.75 μl，去离子水补齐至30 μl。置PE7000仪进行扩增，95℃变性3分钟，然后按95℃×60 s，58℃×30 s，72℃×60 s进行35个循环，最后一个循环72℃延伸5分钟，同时测定其熔点曲线。

3. 统计学处理：用SPSS 10.0软件分析，均用卡方检验。

结 果

1. MS-PCR结果：见表1和表2。在全部45例经亚硫酸氢钠处理的基因组DNA标本中，有8例（其中SCLC 4例；Ⅰ级4例，Ⅱ级3例，Ⅲb级1例）只存在甲基化PCR产物，另外37例标本中都存在非甲基化PCR产物，其中既有甲基化又有非甲基化产物的16例（其中SCLC 2例；Ⅰ级8例、Ⅱ级8例、Ⅲa级0例），只检测出非甲基化产物21例；在23例正常对照组织中，存在甲基化的只有3例（13.04%），两者间比较差异有统计学意义（$\chi^2=10.32$，$P<0.01$）；在年龄、性别、分化程度和肿瘤大小比较无统计学意义（$P>0.05$），在肿瘤组织学分类（SCLC与NSCLC）临床TNM分期比较无显著差异（注：这可能与标本量过少有关，有待进一步证实）。而在相对应的血浆和BALF中分别检出RASSF1A启动子甲基化为13（$\chi^2=15.195$，$P<0.01$）、19（$\chi^2=24.085$，$P<0.01$）例，在肺癌组织甲基化阴性和正常对照的血浆、BALF中均未检测出RASSF1A启动子甲基化，表明三者间有显著的相关性。

2. PCR产物分析：经2%琼脂糖凝胶电泳2小时证实了是一条特异的目的带，见图1。

表1 RASSF1A基因甲基化状况与肺癌病理特征间的关系

特征	例数	肿瘤组织			χ^2
		阳性（例）	阳性率（%）	阴性（例）	
组织学分型					
SCLC	8	6	75.00	2	1.467△
NSCLC	37	18	48.65	19	
临床TNM分型					
Ⅰ级	25	12	48.00	13	0.723*
Ⅱ级	18	11	61.11	7	0.088**
Ⅲa级	2	1	50.00	1	0.003***
性别					
男	28	15	53.57	13	
女	17	9	52.94	8	0.002△△
总计	45	24	53.33	21	

注：*与Ⅱ级比较；**与Ⅰ级比较；***与Ⅲa级比较；△与SCLC比较；△△与男性比较。各指标间比较，P均>0.05

表2 肺癌患者肿瘤组织及其血浆RASSF1A、BALF基因启子异常甲基化检测结果（例）

肿瘤组织	血浆		BALF		合计
	阳性	阴性	阳性	阴性	
阳性	13	11	19	5	24
阴性	0	21	0	21	21

注：M=93bp U=105bp，1～2为血浆，3～4为BALF、5～6为癌组织，8为空白对照，9为分子量标准物

(C)1994-2022 China Academic Journal Electronic Publishing House. All rights reserved. http://www.cnki.net

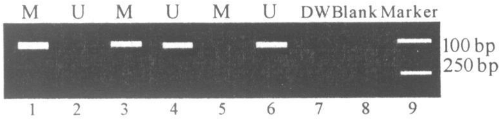

图 1 MS-PCR检测肺癌中 RASSF1A基因甲基化状态电泳图

讨 论

RASSF1A基因全长 1873 bp,包含 2个启动子和 6个外显子,在其启动子区域存在 16个 CpG位点[4]。RASSF1A作为一种肿瘤抑制基因,参与 Ras/RASSF1/ERK通路的信号传导,通过抑制 Ras激活生长效应信号的传导途径,使其丧失抑制生长、促进凋亡和衰老的功能而发挥抑制肿瘤发生的作用,其功能失活将导致对肿瘤的抑制作用丧失。我们实验组用 SYBR Green Ⅰ实时定量 PCR研究了 RASSF1A启动子在 45例肺癌组织及 23例癌旁组织中甲基化化状况,在肺癌组织中,53.33%的病例标本中存在 RASSF1A基因启动子区域 CpG位点甲基化。而正常对照组织中,存在甲基化的只占 13.04%,两者间比较差异有统计学意义(χ^2=10.32, $P<0.01$)。肺癌组织中,RASSF1A基因启动子区域确实存在 CpG位点被甲基化的现象,根据已被广泛证实的启动子区域的 DNA甲基化胞嘧啶的密度和基因转录活性的相互关系[5],可以认为启动子区域 CpG位点甲基化是 RASSF1A失活的主要机制,在肺癌的发生过程中发挥主要作用,并确立 RASSF1A为肺癌的候选抑癌基因。癌与癌旁组织差异显著,但与肿瘤的病理类型、TNM分期年龄性别不相关。

在癌组织甲基化阳性的标本中血浆和 BALF检出率分别达 54.17%、79.17%,血浆中检出阳性率低可能由于癌组织细胞尚无坏死、凋亡或血循环中来自肿瘤细胞的 DNA量太少而低于 MSP检测范围,也有可能是其他有待进一步研究的原因,而 BALF中检出率显著接近于肺癌组织,未检出可能与 BALF中肺癌细胞(包括死亡与凋亡的癌细胞)脱落过少有关,也可能与我们取材有关。而在癌组织甲基化阴性的相应血浆及 BALF中均没检测出 RASSF1A启动子甲基化的存在,说明血浆、BALF和肿瘤组织 RASSF1A甲基化检出率有良好的相关性,由于组织标本不易取得,可以用易得标本(血浆、BALF)早期预测肺癌的发生。

甲基化与非甲基化熔点温度比较发现,甲基化的熔点温度比非甲基化的熔点温度平均高约 3~5度,这是由于基因组 DNA的磺化修饰,MCpG没被转化,而未被甲基化的 CpG被转化,C被转化为 T,熔点温度降低。熔点温度差异不同,证明甲基率(并不是所有的 CpG岛均被甲基化)不同[6]。DNA甲基化是由甲基化酶介导的在 DNA某些碱基上增加一个甲基的化学修饰过程。在肿瘤细胞,DNA甲基化主要发生在启动子区域的 CpG位点,基因由于启动子甲基化而失活,不仅抑制基因的表达,还改变蛋白和 DNA的相互作用,导致染色质结构的改变,是抑制基因活性的一种重要机制。通过最近几年的深入研究,5'-CpG岛的甲基化也被证明是肿瘤抑制基因失活的除突变和等位基因丢失外的第 3种机制,而且在某些情况下是抑癌基因失活的唯一机制[7]。按照 Glen Y等[2]理论,如果甲基化率超过 50%,说明要么 2个等位基因都被甲基化,要么 1个被甲基化,同时另 1个发生杂合性缺失。符合 Knudson AG[8]的"二次打击"学说,RASS1A启动子区域甲基化,是"二次打击"中的"第 1次打击",既而发生"第 2次打击",即未甲基化的等位基因发生杂合性缺失导致抑癌基因 RASSF1A完全失活,失去对细胞异常增殖的抑制调控,促进肺癌的发生。

总之,我们通过检测肺癌组织及其相应血浆、BALF中 RASSF1A基因启动子甲基化,为筛选肺癌新的标志物提供了新的思路,为肺癌的早期诊断近而早期治疗,提高肺癌存活率提供了可能。

参 考 文 献

[1] Gyeong Hoon Kang, Hyeon Joo Lee, Kyu Sang Hwang, et al. Aberrant CpG Island Hypermethylation of Chronic Gastritis in Relation to Aging, Gender, Intestinal Metaplasia, and Chronic Inflammation. American Journal of Pathology, 2003, 163: 1551-1556.

[2] Glen Y, Dammann R, Pfeifer GP. Hypermethylation of the CpG island of Ras association domain family 1A (RASSF1A), a putative tumor suppressor gene from the 3p21.3 locus occurs in a large percentage of human breast cancers. Cancer Res, 2001, 61: 3105-3109.

[3] Herman JG, Graff JR, Myohanen S, et al. Methylation-specific PCR: a novel PCR assay for methylation status of CpG islands. Proc Natl Acad Sci U S A, 1996, 3; 93: 9821-9826.

[4] Dammann R, Li C, Yoon JH, et al. Epigenetic inactivation of a ras association domain family protein fyom the lung suppressor locus 3p21. Nat Genet, 2000, 25: 315-319.

[5] Costell J F, Fruhwald MC, Smiraglia DJ, et al. Aberrant CpG-island methylation has non-random and tumour-type-specific. Nat Genet, 2000, 24: 132-138.

[6] 高云霞,关明,张万忠,等. 胶质瘤患者 RASSF1A基因转录表达和启动子区甲基化的研究. 中华检验医学杂志, 2004, 27: 427-430.

[7] Yuan Y, Mendee R, Sahin A, et al. Hypermethylation leads to silencing of the SYK gene in human breast cancer. Cancer Res, 2001, 61: 5558-5561.

[8] Knudson AG. Two genetic hits (more or less) to cancer. Nat Rev Cancer, 2001, 1: 157-162.

(收稿日期: 2006-06-20)
(本文编辑:李庆宪)

(C)1994-2022 China Academic Journal Electronic Publishing House. All rights reserved. http://www.cnki.net
