# Supplementary material for: Meta-analysis of the accuracy for RASSF1A methylation in bronchial aspirates for the diagnosis of lung cancer
Source: PLoS One. 2024 Jul 25;19(7):e0299447. doi: 10.1371/journal.pone.0299447 (PMC11271935; doi:10.1371/journal.pone.0299447)
Supplement: S1 File — (ZIP) [file pone.0299447.s006.zip › S1 File/Zhang YM 2016.pdf]

肺泡灌洗液中 *SHOX2* 和 *RASSF1A* 基因甲基化联合检测对肺癌的诊断价值

张毅敏,王明丽,吴　杰,单绿虎,张剑英,熊　娟,徐笑红
(浙江省肿瘤医院,浙江 杭州 310022)

摘　要:[目的]评估肺泡灌洗液(BALF)中 *SHOX2* 和 *RASSF1A* 基因甲基化联合检测(任意阳性)在肺癌诊断中的临床价值。[方法]收集经病理确诊的 580 例患者,其中肺癌 261 例,良性肺部疾病 293 例, 非肺部的恶性肿瘤 26 例。将良性肺部疾病和非肺部的恶性肿瘤患者共 319 例作为对照组。对全部 580 例患者的 BLAF 样本,采用实时荧光 PCR(RT-PCR)与测序法检测 BALF 中 *SHOX2* 和 *RASSF1A* 两种基因甲基化情况,比较两种检测方法的一致性。以病理诊断为金标准,对 277 例具有细胞学结果的患者(肺癌组 130 例,对照组 147 例),比较采用 RT-PCR 法 *SHOX2* 和 *RASSF1A* 基因甲基化检测与细胞学检测对肺癌诊断的敏感性和特异性。[结果]RT-PCR 法与测序法两种方法检测 580 例患者 BLAF 样本 *SHOX2* 基因和 *RASSF1A* 基因甲基化结果的一致率分别是 97.5%和 98.0%。580 例 BLAF 样本采用 RT-PCR 法检测 *SHOX2* 和 *RASSF1A* 基因甲基化任意阳性对肺癌诊断的敏感性和特异性分别是 74.3%和 87.1%。在 277 例有细胞学结果的患者中,RT-PCR 法 *SHOX2* 和 *RASSF1A* 基因甲基化检测任意阳性对肺癌诊断的敏感性和特异性分别为 83.2%和 92.5%,与细胞学检测(44.6%和 98.6%)相比,敏感性显著提高,但特异性有所降低,差异均有统计学意义($P<0.01$,$P<0.05$)。基因甲基化联合检测(任意阳性)阳性率在细胞学发现癌细胞组为 98.2%(57/58),结果未明组为 100.0%(35/35),未发现癌细胞组为 37.8%(14/37)。[结论]采用 RT-PCR 法进行 BALF 中 *SHOX2* 和 *RASSF1A* 基因甲基化联合检测,可以作为肺癌细胞学检查的补充,特别是在细胞学检测结果未明或阴性时。

主题词:*SHOX2*;*RASSF1A*;基因甲基化;肺泡灌洗液;肺肿瘤
中图分类号:R734.2　　**文献标识码**:A　　**文章编号**:1671-170X(2016)12-1032-05
doi:10.11735/j.issn.1671-170X.2016.12.B010

Combined Detection of *SHOX2* and *RASSF1A* Gene Methylation in Bronchoalveolar Lavage Fluid for Diagnosis of Lung Cancer

ZHANG Yi-min,WANG Ming-li,WU Jie,et al.
(*Zhejiang Cancer Hospital,Hangzhou* 310022,*China*)

Abstract:[Objective] To evaluate the application of combined detection of *SHOX2* and *RASSF1A* gene methylation in bronchoalveolar lavage fluid (BALF) for diagnosis of lung cancer. [Methods] BLAF samples and the pathological data were collected from 261 patients with lung cancer and 319 controls. The methylation statues of *SHOX2* and *RASSF1A* genes in BALF were detected by quantified real-time PCR(RT-PCR) and sequencing,and the consistency of two methods was analyzed. The sensitivity and specificity of either positive *SHOX2* or *RASSF1A* methylation for diagnosis of lung cancer were compared with cytological examination in 277 patients with cytological results (130 cases in lung cancer group,147 controls) .[Results] The consistency rates of *SHOX2* and *RASSF1A* gene methylation detected by RT-PCR and sequencing were 97.5% and 98.0% ,respectively. The sensitivity and specificity of combined positive *SHOX2* and *RASSF1A* methylation for diagnosis of lung cancer in 580 BALF samples detected by RT-PCR were 74.3% and 87.1%,respectively. In 277 cases with cytological results,the sensitivity of combined detection of *SHOX2* and *RASSF1A* methylation for diagnosis of lung cancer detected by RT-PCR was 83.2% ,higher than that in cytological detection(44.6%,$P<0.01$); and the specificity in gene methylation(92.5%) was lower than that in cytological detection(98.6%,$P<0.05$). The positive rate of combined *SHOX2* and *RASSF1A* gene methylation was 98.2%(57/58)in cytology positive group,100.0%(35/35) in cytology undetermined group, and 37.8%(14/37)in cytology negative group. [Conclusion] Combination detection of methylation of *SHOX2* and *RASSF1A* genes in BALF by RT-PCR may be used as a supplement to cytology for diagnosis of lung cancer,especially in patients with undetermined or negative cytology.

Subject words:*SHOX2*;*RASSF1A*;gene methylation;bronchoalveolar lavage fluid;lung neoplasms

2010 年我国第 3 次全国死因分析显示,我国肺癌发病率和死亡率都居恶性肿瘤首位[1]。与其他恶性肿瘤相比,由于缺乏有效的筛查手段,肺癌的早期诊断率大大低于乳腺癌、前列腺癌和结直肠肿癌,这是其高死亡率的原因之一[2]。近年来在纤维支气管

基金项目:浙江省医药卫生科技项目(2014KYB043;2016KYB034)
通讯作者:徐笑红,主任医师,学士;浙江省肿瘤医院检验科,浙江省杭州市拱墅区半山东路 1 号(310022);E-mail:zjhzxxh@163.com
收稿日期:2016-09-17;**修回日期**:2016-10-28

镜应用基础上发展起来的支气管肺泡灌洗技术(BAL)为呼吸系统病变的诊断和疗效观测开辟了一条新途径。它可获得纤维支气管镜所不能探及的细胞学标本以提高癌细胞的检出率。然而,细胞学检测存在受人为因素影响大和低敏感性(43%~48%)的问题[3]。DNA 甲基化作为一种新型的分子标志物,已经被应用在改善肺癌诊断效力上[4,5]。

矮小同源盒基因 (short stature homobox2, *SHOX2*) 是目前在肺癌诊断中应用最广泛的甲基化指标,但随着研究和应用的深入,发现其对肺腺癌的诊断敏感性显著低于对小细胞肺癌和肺鳞癌[6,7]。Ras 相关区域家族 1A(ras-association domain family 1 A,*RASSF1A*),作为一种新型肿瘤抑制基因,其在肿瘤发生及发展过程中的作用尤为重要。Shimizu 等[8]证实了 *RASSF1A* 的表达缺失与肺腺癌的发生密切相关。Tomizawa 等[9]还提出,在非小细胞肺癌(NSCLC)中,*RASSF1A* 在腺癌中的甲基化频率比在鳞癌中更高。在本研究中,我们联合检测 *SHOX2* 和 *RASSF1A* 基因在 BALF 中的甲基化状态以评估其在肺癌辅助诊断中的临床价值。同时,比较基因甲基化检测与常规细胞学检测对肺癌的诊断效能。

1 资料与方法

1.1 研究对象

收集 580 例患者的 BALF 及临床病理资料,其中肺癌组 261 例(腺癌 113 例,鳞癌 56 例,大细胞肺癌 3 例,小细胞肺癌 38 例,组织类型不明确肺癌 51 例)。对照组 319 例(良性肺部疾病 293 例,非肺部的恶性肿瘤 26 例)。其中 277 例(肺癌组 130 例,对照组 147 例)具有细胞学结果。对 20 例(共 21 例,1 例未做随访)甲基化检测阳性但细胞学阴性的临床诊断为肺部感染、支气管扩张、淀粉样变等良性病例进行随访(每年 1 次,为期 5 年)。入组患者的临床病理特征详见表 1(Table 1)。之前的一项基于 BALF 的研究表明,性别和年龄对 *SHOX2* 水平没有影响[6],因此,本研究在分析肺癌组和对照组时不考虑性别和年龄配对。

1.2 主要试剂和仪器

人 *SHOX2*、*RASSF1A* 基因甲基化 DNA 检测试剂盒(实时荧光 PCR 法)由上海透景生命科技股份有限公司提供;实时荧光 PCR 检测系统为 ABI7500;DNA 提取试剂盒购自天根生化科技有限公司生产的血液/细胞/组织基因组 DNA 提取试剂盒;亚硫酸盐修饰试剂盒购自 ZYMO RESEARCH 生物公司 EZ DNA Methylation-Direct™ kit。

1.3 DNA 提取与亚硫酸盐修饰

取肺泡灌洗液 10ml,10 000rpm 离心 5min,去除上清,沉淀用于 DNA 提取,具体抽提步骤参见天根生化科技有限公司生产的血液/细胞/组织基因组 DNA 提取试剂盒说明书。提取后的 DNA 直接进行亚硫酸盐修饰,具体步骤参见 ZYMO RESEARCH 生物公司 EZ DNA Methylation-Direct™ kit 说明书。

1.4 实时荧光 PCR(RT-PCR)检测 *SHOX2*、*RASSF1A* 基因甲基化

修饰后的 DNA 直接用人 *SHOX2*、*RASSF1A* 基因甲基化 DNA 检测试剂盒(RT-PCR 法)检测,具体步骤与结果判断方法见上海透景生命科技股份有限公司说明书。

1.5 测序检测 *SHOX2*、*RASSF1A* 基因甲基化

根据修饰后的 DNA 序列,设计 *SHOX2* 基因和 *RASSF1A* 基因测序引物,序列如下:*SHOX2* 基因测序引物:F3:GGTGTTGTGTCGTATAGGGAGT,R3:TCCGCCTCCTACCTTCTAAC;*RASSF1A* 基因测序引物:F11:GAGGGAAGGAAGGGTAAGG,R11:GAGGG-AAGGAAGGGTAAGG。

测序 PCR 体系(40μl):修饰后 DNA 5μl,上游引物 (10μmol/L)0.8μl,下游引物(10μmol/L)0.8μl,2*Taq 缓冲液 (含 dNTPs 和 Taq 酶)20μl,加水 13.4μl。

PCR 扩增程序:预变性 95℃ 10mim;循环 95℃ 30s,58℃ 35s,72℃ 30s,45 个循环;延伸 72℃ 8min。

Table 1 Clinical features of the patients in lung cancer group and control group(%)

Items	Total	Lung cancer group	Control group
Age(years old)	580(100.0)	261(100.0)	319(100.0)
≤50	141(24.3)	33(12.7)	108(33.9)
51~60	172(29.6)	76(29.1)	82(25.7)
>60	267(46.1)	152(58.2)	129(40.4)
Medium	60.0	61.5	58.0
Range	10~88	24~86	10~88
Gender			
Male	391(67.4)	195(74.7)	197(61.8)
Female	189(32.6)	66(25.3)	122(38.2)

PCR 产物经检测合格后,送上海生工生物工程有限公司测序。

1.6 BALF 细胞学检测

首先在要灌洗的肺段经活检孔注 2%利多卡因 1~2ml 行灌洗肺段局部麻醉；然后经活检孔 37℃灭菌生理盐水 25~50ml,立即用负压吸回灌洗液,装入容器中送检。

1.7 统计学处理

以病理诊断作为金标准,计算肺癌甲基化检测用于肺癌诊断的敏感性与特异性,比较细胞学检测方法与肺癌甲基化检测诊断肺癌的敏感性与特异性。

采用 SPSS22.0 统计学软件对数据统计分析,采用卡方检验判断组间是否存在显著性差异，使用 *Kappa* 系数分析 RT-PCR 与测序方法检测甲基化一致性,并计算 *Kappa* 系数 95%可信区间(95%CI)。$P<0.05$,认为差异具有统计学意义。

2 结 果

2.1 RT-PCR 法与基因测序检测 *SHOX2*、*RASSF1A* 甲基化一致性研究

对 580 例 BALF 样本,分别用 RT-PCR 和基因测序的方法检测其 *SHOX2*、*RASSF1A* 甲基化情况。结果显示,两种方法学对 *SHOX2* 甲基化检测一致率为 97.5%(*Kappa*=0.946,95% CI:0.918~0.973)，对 *RASSF1A* 甲基化检测一致率为 98.0%(*Kappa* =0.951,95% CI:0.923 ~ 0.978)(Table 2)。

Table 2 Consistency of *SHOX2* and *RASSF1A* gene methylation detection by RT-PCR and gene sequencing

Genes	RT-PCR	N	Sequencing		*Kappa*	*Kappa* coefficient(%)	95%CI
			Positive	Negative			
SHOX2	Positive	202	196	6	0.946	97.5	0.918~0.973
	Negative	378	9	369			
	Total	580	205	375			
RASSFIA	Positive	159	152	7	0.951	98.0	0.923~0.978
	Negative	421	5	416			
	Total	580	157	423			

2.2 RT-PCR 法检测 *SHOX2* 和 *RASSF1A* 基因甲基化在肺癌辅助诊断中的临床价值

580 例样本采用 RT-PCR 法检测 *SHOX2* 和 *RASSF1A* 基因甲基化任意阳性对肺癌诊断的敏感性和特异性分别是 74.3%和 87.1%。按不同病理诊断类型分组,RT-PCR 法检测 *SHOX2*、*RASSF1A* 基因甲基化结果如表 3(Table 3)所示。其中肺癌组中,*SHOX2* 和 *RASSF1A* 基因甲基化单独阳性、双阳性和任意阳性的阳性率分别为 69.7%、53.2%、49.4%和 74.3%，明显高于对照组的 10.6%、5.9%、3.7%和 12.8%,其差异具有统计学意义($P<0.01$)。

与 *RASSF1A* 相比,在各肺癌病理亚组,*SHOX2* 甲基化阳性率更高,依次是:大细胞肺癌(100.0%,3/3),小细胞肺癌(94.7%,36/38),肺鳞癌(85.7%,48/56)，组织类型不明确肺癌（68.6%,35/51)，腺癌(53.1%,60/113)。

2.3 RT-PCR 法基因甲基化检测与细胞学检测肺癌的诊断效能

277 例样本(肺癌组 130 例,对照组 147 例),同时用细胞学方法和基因甲基化检测法(RT-PCR)分析（Table 4)，肺癌组中，细胞学发现癌细胞 58 例(44.6%);基因甲基化法(任意阳性)106 例(81.5%)。

Table 3 Methylation status of *SHOX2* and *RASSF1A* genes in different pathological diagnosis specimens[n(%)]

Groups	Pathology	N	*SHOX2*	*RASSFIA*	Both positive	Either positive
Lung cancer	Adenocarcinoma	113	60(53.1)	49(43.3)	39(35.5)	70(61.9)
	Squamous cell carcinoma	56	48(85.7)	25(44.6)	25(44.6)	48(85.7)
	Large cell carcinoma	3	3(100.0)	2(66.6)	2(66.6)	3(100.0)
	Small cell carcinoma	38	36(94.7)	36(94.7)	36(94.7)	37(97.3)
	Undetermined type	51	35(68.6)	27(52.9)	27(52.9)	36(70.5)
	Total	261	182(69.7)	139(53.2)	129(49.4)	194(74.3)
Controls	Benign lung disease	293	31(10.5)	17(5.8)	10(3.4)	38(12.9)
	Non-lung malignancies	26	3(11.5)	2(7.6)	2(7.6)	3(11.5)
	Total	319	34(10.7)	19(6.0)	12(3.8)	41(12.9)

对照组中，细胞学未发现癌细胞 145 例(98.6%)，基因甲基化法(双阴性)124 例(84.3%)。基因甲基化检测相对于细胞学可显著提高肺癌诊断敏感性（从 44.6%到 81.5%），但特异性从 98.6%降到 84.3%。对 20 例(共 21 例，1 例未做随访)甲基化检测阳性但细胞学阴性的临床诊断为肺部感染、支气管扩张、淀粉样变等良性病例进行随访(每年 1 次，为期 5 年)，共发现肺癌 13 例。根据此数据，联合基因甲基化检测的敏感性和特异性分别调整为 83.2%(119/143)和 92.5%(124/134)，与细胞学检测的敏感性和特异性相比，敏感性提高显著，但特异性有所降低，均有统计学意义(P<0.01，P<0.05)。

绘制柱状图描述不同细胞学结果中基因甲基化检测情况(Figure 1)，结果分析显示，基因甲基化联合检测(任意阳性)阳性率在细胞学发现癌细胞组为 98.2%(57/58)，结果未明组为 100.0%(35/35)，未发现癌细胞组为 37.8%(14/37)。提示采用 RT-PCR 法进行 SHOX2 和 RASSF1A 基因甲基化联合检测(任意阳性)可以作为细胞学检测的补充，特别是在细胞学检测结果未明或阴性时。

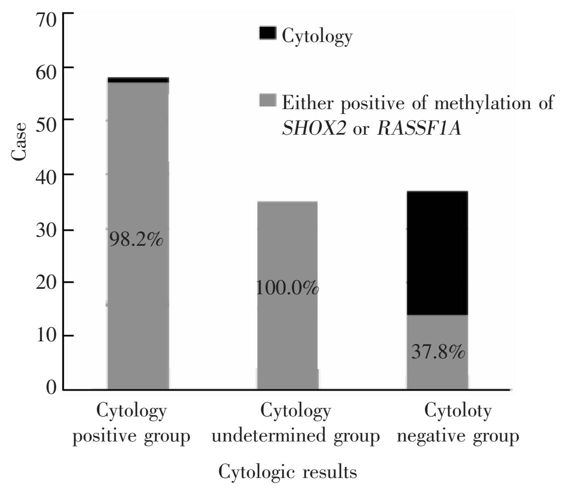

Figure 1　Methylation status of *SHOX2* gene and *RASSF1A* gene in different cytological diagnosis results

Table 4　Comparison of the efficacy of *SHOX2* and *RASSF1A* gene methylation and cytology in the diagnosis of lung cancer

Methods	Sensitivity(%)	Specificity(%)
Cytology	44.6(58/130)	98.6(145/147)
RASSF1A	60.7(79/130)	92.5(136/147)
SHOX2	75.3(98/130)	86.3(113/147)
SHOX2 or *RASSF1A*	81.5(106/130)	84.3(124/147)
Cytology or *SHOX2* or *RASSF1A*	82.3(107/130)	84.3(124/147)

3　讨　论

DNA 甲基化作为一种新型的分子标志物，在肿瘤的发生、发展中发挥重要的作用，目前已经应用在改善肺癌诊断效力[4,5]上。2010 年，Schmidt 等[6]人首次发现 *SHOX2* 甲基化能较好地区分肺部良恶性病变，敏感性和特异性分别为 68%和 95%。他们继续深入研究肺癌患者血液[10]和组织[11]中 *SHOX2* 基因的甲基化，结果显示甲基化的 *SHOX2* 不仅可以作为早期检测的生物指标，而且可以作为 NSCLC 预后的独立预测指标[12]。但随着研究的深入，发现 *SHOX2* 甲基化对肺腺癌的诊断敏感性（47%~65%）显著低于小细胞肺癌(82%~97%)和肺鳞癌(81%~82%)[6,7]。*RASSF1A* 作为一种新型肿瘤抑制基因，其在肿瘤发生及发展过程中的作用尤为重要。Shimizu 等[8]证实了 *RASSF1A* 基因的表达缺失与肺腺癌的发生密切相关。Tomizawa 等[9]还提出，在 NSCLC 中，*RASSF1A* 在腺癌中的甲基化频率比在鳞癌中更高。目前国内还没有两者联合检测对肺部疾病诊断和鉴别诊断的大规模数据。我们对用 RT-PCR 法检测 580 例患者的 BALF 中 *SHOX2*、*RASSF1A* 甲基化结果进行统计分析，评价其对肺癌诊断的临床价值。

研究甲基化的方法有多种，本研究所选用的 RT-PCR 的方法结合了亚硫酸盐修饰和 Taqman 探针两种技术。利用亚硫酸盐使未甲基化的 C 碱基转换为 U 碱基，在 PCR 扩增过程中，再转换为 T 碱基，而甲基化的 C 碱基保持不变，因此可以区分甲基化和未甲基化的 C 碱基；针对修饰前后的序列差异用特异性软件设计甲基化引物进行 PCR 扩增；利用 Taqman 探针对扩增产物进行检测，通过优化的反应体系和高特异 Taq 酶的使用，在实时荧光 PCR 平台上实现对样品中甲基化 DNA 的特异检测。580 例样本同时采用 RT-PCR 和测序方法检测 *SHOX2*、*RASSF1A* 的甲基化状态，一致率分别是 97.5%和 98.0%。

在纤维支气管镜应用的同时取得 BALF 是临床的常规操作。选用 BALF 的优点是取材特异，不易混入其他肿瘤细胞，且肿瘤细胞数量相对较多。在本研究中，肺癌样本 261 例(45.0%)，良性肺部病变 293 例(50.5%)，另特别收集 26 例其他非肺部肿瘤样本(4.5%)。*SHOX2* 和 *RASSF1A* 基因甲基化在肺癌组

明显高于对照组(P<0.001),可以有效鉴别良恶性肺部疾病以及肺癌与其他部位肿瘤。

在对肺癌亚组分析中发现,*SHOX2* 基因甲基化在大细胞肺癌中阳性率最高(100.0%),依次为小细胞肺癌(94.7%)、鳞癌(85.7%)、腺癌(53.1%),各组之间差异有统计学意义(P<0.001)。对这种现象目前没有明确的解释，肿瘤的位置是一种可能的影响因素,小细胞肺癌和鳞癌发生的部位居中,支气管镜比较容易到达,取得目标 DNA 的数量和可能性都比周边位置高[6]。

在肺腺癌组中，两基因单独检测的检出率均不高,*RASSF1A* 基因为 43.3%(49/113),*SHOX2* 基因也仅有 53.1%(60/113)，且 *SHOX2* 和 *RASSF1A* 基因的双阳性样本更少(35.5%,39/113),但两者联合检测可使基因甲基化对肺腺癌的检测敏感性提高到 61.9%(70/113,P<0.05)。*SHOX2* 和 *RASSF1A* 基因在不同肺腺癌标本中出现阳性，可能预示两者甲基化的调控机制以及肺腺癌的形成机制会有所不同。

BALF 常规细胞形态学检查作为评价肺部疾病的重要手段已广泛应用于临床,然而,细胞学检测存在受人为因素影响大和低敏感性(43%~48%)的问题[3],同时还会出现大量结果未明的检测报告。在本研究中,277 例样本同时进行了细胞学和甲基化检测，肺癌组中细胞学检测发现癌细胞 58 例,基因甲基化检测阳性率 98.2%;结果未明 35 例,基因甲基化检测阳性率 100.0%;未发现癌细胞 37 例,基因甲基化检测阳性率 37.8%。联合基因甲基化相对于细胞学可显著提高肺癌诊断敏感性(从 44.6%到 81.5%)。但以细胞学检测结果为基础计算的联合基因甲基化检测特异性仅为 84.3%，与文献报道的 95%~98%[6,7]有较大差异。对 20 例(共 21 例,1 例未做随访)甲基化检测阳性但细胞学阴性的临床诊断为肺部感染、支气管扩张、淀粉样变等良性病例进行随访（每年 1 次,为期 5 年),经随访后,共发现肺癌 13 例。根据此数据，调整联合基因甲基化检测的敏感性和特异性分别为 83.2%(119/143)和 92.5%(124/134)。联合基因甲基化作为一种新型的肺癌辅助诊断方法，特别是在细胞学结果未明或阴性时可显著提高对肺癌的检出率。

在之后的研究中，为更加有效地评估甲基化检测在肺癌早期诊断和预后评估中的诊断效力，研究还将纳入不同肺癌分期和术后的样本，

总之,RT-PCR 方法检测肺泡灌洗液中 *SHOX2* 和 *RASSF1A* 基因甲基化状态具有高敏感性和高特异性。两者联合检测是非常有潜力的肺癌诊断的新型标志物,可以作为细胞学检测的补充,特别是在细胞学检测结果未明或阴性时。

参考文献：

[1] Chen WQ,Zhang SW,Zeng HM,et al.Report of cancer incidence and mortality in China,2010 [J].China Cancer,2014,23(1):1-10.[陈万青,张思维,曾红梅,等,中国 2010 年恶性肿瘤发病率和死亡率[J].中国肿瘤,2014,23(1):1-10.]

[2] Jemal A,Siegel R,Ward E,et al.Cancer statistics,2007[J].CA Cancer J Clin,2007,57:43-66.

[3] Schreiber G,McCrory DC.Performance characteristics of different modalities for diagnosis of suspected lung cancer:summary of published evidence[J].Chest,2003,123:115-128.

[4] Anglim PP,Galler JS,Koss MN,et al.Identification of a panel of sensitive and specific DNA methylation markers for squamous cell lung cancer[J].Mol Cancer,2008,7:62.

[5] Ehrich M,Field JK,Liloglou T,et al.Cytosine Methylation profiles as a molecular marker in non-small cell lung cancer[J].Cancer Res,2006,66:10911-10918.

[6] Schmidt B,Liebenberg V,Dietrich D,et al.SHOX2 DNA methylation is a biomarker for the diagnosis of lung cancer based on brochial aspirates[J].BMC Cancer,2010,10:600.

[7] Ilse P,Biesterfeld S,Pomjanski N,et al.Analysis of SHOX2 methylation as an aid to cytology in lung cancer diagnosis[J]. Cancer Genom Proteom,2014,11:251.

[8] Shimizu K,Kato A,Hinotsume D,et al.Reduced expressions of Foxpl and RASSF1A genes in lung adenocarcinomas induced by N-nitrosobis (2-hydroxypropyl)amine in rats[J].Cancer Lett,2006,236:186-190.

[9] Tomizawa Y,Iijima H,Nomoto T,et al.Clinicopathological significance of aberrant methylation of RARbeta2 at 3p24,RASSF1 A at 3p21.3,and FHIT at 3p14.2 in patients with non-small cell lung cancer [J]. Lung Cancer,2004,46:305-312.

[10] Kneip C,Schmidt B,Seegebarth A,et al. SHOX2 DNA methylation is a biomarker for the diagnosis of lung cancer in plasma[J].Thorac Oncol,2011,6:1632-1638.

[11] Dietrich D,Kneip C,Raji O,et al.Performance evaluation of the DNA methylation biomarker SHOX2 for the aid in diagnosis of lung cancer based on the analysis of bronchial aspirate[J].Int J Oncol,2011,40(3):825-832.

[12] Dietrich D,Hasinger O,Liebenberg V,et al. DNA methylation of the homebox genes PITX2 and SHOX2 predicts outcome in non-small-cell lung cancer patients [J]. Diagn Mol Pathol,2012,21(2):93-104.
